# A Novel Low-Cost Phantom for Ultrasound-Guided Fascia Iliaca Nerve Blocks

**DOI:** 10.5070/M5.52321

**Published:** 2026-04-30

**Authors:** Laura Nolting, Heather A Brown

**Affiliations:** * Prisma Health Midlands, Department of Emergency Medicine, Columbia, SC

## Abstract

**Audience:**

This phantom is designed to instruct emergency medicine residents in ultrasound-guided fascia iliaca (FI) nerve blocks but could also be used for medical students and attending physicians.

**Introduction:**

Ultrasound-guided regional nerve blocks are increasingly used in the emergency department for pain management. The FI block, used for pain management in patients with hip fractures, have been shown to provide pain relief for up to eight hours with rare complications and to decrease need for parenteral pain medication.^1^,^2^ It is considered best practice to train physicians in ultrasound-guided procedures such as FI blocks by using phantoms, which are objects designed to mimic human tissue and anatomy relevant to the specific ultrasound-guided procedure. Commercial trainers are available but are quite expensive with those designed to mimic femoral anatomy priced at or over $5,000 USD, and they still lack some of the anatomical landmarks useful in FI blocks.^3^,^4^ Several lower cost FI models have been described made from a variety of perishable items including gelatin, tofu, chicken, konnyaku jelly, pork and meat glue.^5^–^10^ However, these models have the downside of limited uses due to the nature of perishable materials, and most are also lacking in block specific landmarks and durability.

**Educational Objectives:**

By the end of the training session using the FI phantom and bedside ultrasound, learners should be able to: 1) discuss indications, contraindications, and complications of FI blocks; 2) identify anatomy relevant to performing an FI block on ultrasound; and 3) independently perform an FI block or demonstrate proper needle position for FI block on ultrasound of the phantom.

**Educational Methods:**

This low-cost FI block phantom was developed using ballistics gel to create the fascia layers and muscles, bungee cord for nerve, and latex balloons for vessels. Ballistics gel was also used as the base medium for the phantom.

**Research Methods:**

Participants completed a short electronic survey following the educational session using five-point Likert scale questions to evaluate the phantom based on ultrasound image quality, anatomical accuracy, and perceived durability. An additional question asked participants if they felt more confident performing an FI block after practicing on the phantom.

**Results:**

Twenty-four emergency medicine residents completed the training session and the post-training survey. All learners were able to successfully demonstrate proper needle placement on ultrasound for the FI block. On a five-point Likert scale, ranging from 1 (very poor) to 5 (excellent), participants rated the phantom in durability, anatomical accuracy, and ultrasound image quality. Most participants agreed that the phantom was anatomically accurate (median 4) and durable (median 4). The phantom performed the best in the category of ultrasound image quality (median 5). Most participants agreed that practicing with the phantom increased their confidence in performing FI blocks (median 4). The phantom held up to 100 needle sticks with only mild degradation in image quality.

**Discussion:**

The phantom presented here effectively taught EM residents proper needle placement for an FI block since all participants were able to demonstrate appropriate needle placement. The phantom was low cost, particularly compared to commercial trainers, and held up to a large number of needle sticks.[Fig f8-jetem-11-2-i43]

**Topics:**

Nerve blocks, ultrasound phantom, pain management, hip fractures, point of care ultrasound.

## USER GUIDE

**Table:** 

List of Resources:	
Abstract	43
User Guide	45
Appendix A: PowerPoint Presentation	50

**Learner Audience:**

Medical Students, Interns, Junior Residents, Senior Residents, EM Attendings

**Time Required for Implementation:**

Creation of the model takes about an hour and a half.

Learners will spend about five minutes discussing the indications, contraindications, and complications of fascia iliaca (FI) blocks with the instructor. Each learner should spend about five minutes orienting themselves with the ultrasound anatomy and placing the needle in the appropriate position for an FI block. Each learner should be able to simulate appropriate needle position three times in a five-minute period. A three-to-one learner to instructor ratio would require a minimum of 20 minutes to complete the activities.

**Recommended Number of Learners per Instructor:**

A maximum of three learners to one instructor is recommended per phantom. Higher learner to instructor ratios can be used if needed, allotting approximately five additional minutes for each learner.

**Topics:**

Nerve blocks, ultrasound phantom, pain management, hip fractures, point of care ultrasound.

**Objectives:**

By the end of the training session using the FI phantom and bedside ultrasound, learners should be able to:

1. Discuss indications, contraindications, and complications of FI blocks
2. Identify anatomy relevant to performing an FI block on ultrasound
3. Independently perform an FI block or demonstrate proper needle position for FI blocks on ultrasound of the phantom

### Linked objectives and methods

Ultrasound guided nerve blocks are a useful pain management adjunct in the emergency department (ED) which can improve pain scores while decreasing the need for opiate pain medications.^11^,^12^ The FI block is particularly useful in the ED for patients with hip fractures because it provides up to eight hours of pain relief and has a low complication rate.^1^,^2^ This skill is particularly timely for emergency medicine (EM) residents since the Accreditation Council for Graduate Medical Education (ACGME) announced proposed updates in February of 2025 to include requiring regional anesthesia for procedural competency. Also, the Council for Residency Directors in Emergency Medicine (CORD) includes demonstrating knowledge of principles of regional anesthesia into their model curriculum.^13^,^14^ While EM residents are well trained in point of care ultrasound, the FI block relies on unique landmarks that EM residents may not be familiar with. Commercial trainers are available but are quite expensive with those designed to mimic femoral anatomy priced at or over $5,000 USD, and they still lack some of the anatomical landmarks useful in FI blocks including the fascial layers and muscles.^3^,^4^ Lower cost perishable models from a variety of materials (including gelatin, tofu, chicken, konnyaku jelly, pork and meat glue) have been described but are also lacking in block specific landmarks (sartorius and iliaca muscles) and durability.^5^–^10^ We sought to create a FI block model that was highly realistic, incorporating all anatomical landmarks used in the block, while using nonperishable, durable materials so that the phantom could be used recurrently.

### Recommended pre-reading for instructor

- Atchabahain A, Leunen I, Vandepitte C, Lopez A. Ultrasound-guided Fascia Iliaca nerve block. New York School of Regional Anesthesia (NYSORA). Accessed May 1, 2025. https://www.nysora.com/topics/regional-anesthesia-for-specific-surgical-procedures/lower-extremity-regional-anesthesia-for-specific-surgical-procedures/ultrasound-guided-fascia-iliaca-block/
- O’Reilly N, Desmet M, Kearns R. Fascia iliaca compartment block. *BJA Education*. 2019:19(6);191–197.
- Fascia iliaca blocks for hip fractures. Highland EM Ultrasound. Accessed May 1, 2025. https://highlandultrasound.com/new-blog/2014/8/8/fascia-iliaca-block-for-hip-fractures

### Learner responsible content (LRC)

- Atchabahain A, Leunen I, Vandepitte C, Lopez A. Ultrasound-guided Fascia Iliaca nerve block.” New York School of Regional Anesthesia (NYSORA). Accessed May 1, 2025. https://www.nysora.com/topics/regional-anesthesia-for-specific-surgical-procedures/lower-extremity-regional-anesthesia-for-specific-surgical-procedures/ultrasound-guided-fascia-iliaca-block/
- O’Reilly N, Desmet M, Kearns R. Fascia iliaca compartment block. *BJA Education*. 2019:19(6);191–197.
- Fascia iliaca blocks for hip fractures. Highland EM Ultrasound. Accessed May 1, 2025. https://highlandultrasound.com/new-blog/2014/8/8/fascia-iliaca-block-for-hip-fractures

### Implementation methods

This model should ideally be used with a learners-to-faculty ratio of 3:1. Any ultrasound machine with a linear probe can be used. Faculty should review the phantom landmark ultrasound images with the learners and demonstrate proper FI technique and needle placement for the block. At least once, each learner should attempt appropriate needle placement under faculty supervision with real-time feedback. Faculty should facilitate discussion of FI block indications, contraindications, and complications during the session.

### List of items required to replicate this innovation

1. Ballistics gel: Ranges from $6.60 – $43 per pound depending on amount and grade purchased: Forty-three dollars for one pound of medical grade to low end of $6.60 per pound if buying in bulk (17-pound block available for $114 on Amazon.) We purchased a 6.6-pound block of ballistics gel from Amazon for $70 (and used two pounds at $21.20.)
2. Bungee Cord ($3.19 on Amazon)
3. Long latex balloons ($5.50 for pack of 100 on Amazon) or ¼ inch latex rubber tubing ($6.89 on Amazon)
4. Plastic food storage container 6.75 in (L) × 4.75 in (W) × 2.25 in (H) or similar dimensions ($3.00 each on Amazon)
5. All-purpose flour ($2.00 for 5-pound bag)
6. Additional household items needed:
7. Scissors
8. Two or three oven safe glass dishes for melting ballistics gel

### Approximate cost of items to create this innovation

Cost depends on the type and amount of ballistics gel purchased. We spent $32.89 on this phantom by purchasing a 6.6 pound block of ballistics gel from Amazon for $70 (and used two pounds at $21.20.)

### Detailed methods to construct this innovation

1. Melt two pounds of ballistics gel in a glass container. Ballistics gel can be melted in the oven, crockpot or stove top, but should be heated below the 325°F flashpoint.
2. Measure a long latex balloon or ¼ inch latex tubing to fit the width of the container. Fill the balloon or tubing with water and tie it off.
3. Cut the bungee cord to fit the width of the container.
4. Pour a small amount of melted ballistics gel into another oven safe container to cover the bottom (about 1/16 of an inch) in order to make a thin fascial layer. Add ¼ teaspoon of flour to the ballistics gel in the container and stir. The gel will begin to stiffen and take on a slightly white color. Split the contents in half and place ½ in each of two glass containers around the same size as your phantom container. Alternatively, you can use one larger container and cut the fascial layer in half once it has cooled. Reheat until the gel has melted in the bottom of the dishes again creating your two fascial layers.[Fig f1-jetem-11-2-i43]
5. When the rest of the ballistics gel is cool enough to touch but not yet solidified, take a golf ball sized amount and mold it into an oval-shaped, flat, muscle-like structure. Repeat this process so that you have two muscles (sartorius and iliacus).[Fig f2-jetem-11-2-i43]
6. Pour a small amount of your melted ballistics gel into the bottom of your phantom container (just enough to cover the bottom). You can use a toothpick to break up air bubbles in the melted ballistics gel at each step to improve image quality.[Fig f3-jetem-11-2-i43]
7. Place your iliacus muscle on one side of the container and the bungee cord beside it.[Fig f4-jetem-11-2-i43]
8. Pour more ballistics gel into the container to cover the structures in the bottom.[Fig f5-jetem-11-2-i43]
9. Place a fascial layer (fascia iliaca) over the hot gel, gently pressing down to express any air pockets.
10. Place the sartorius muscle directly on top of the fascia iliaca overlying the iliaca muscle. Place your vessels on the fascia iliaca just to the right of the femoral nerve.[Fig f6-jetem-11-2-i43]
11. Pour more melted gel on top to just cover the structures and then place the second fascial layer (fascia lata). Again, gently press to remove any air pockets.[Fig f7-jetem-11-2-i43]
12. Pour the remainder of the melted gel on top to fill the container to the top.

### Results and tips for successful implementation

This phantom was used for a training session during the weekly EM resident didactic session in February of 2024. Twenty-four EM residents completed the training session and the post-training survey. Seven participants were first year residents, ten were in their second year, and seven were in their third and final year of training. Thirteen (54%) participants had not previously performed an FI block, and the remaining eleven participants had experience ranging from one to four previous FI blocks. All learners were able to successfully demonstrate proper needle placement on ultrasound for the FI block. On a five-point Likert scale, ranging from 1 (very poor) to 5 (excellent), participants rated the phantom in durability, anatomical accuracy, and ultrasound image quality. Most participants agreed that the phantom was anatomically accurate (median 4) and durable (median 4). The phantom performed the best in the category of ultrasound image quality (median 5). Most participants agreed that practicing with the phantom increased their confidence in performing FI blocks (median 4). The phantom held up to 100 needle sticks with only mild degradation in image quality. Participants did not inject into the phantom because doing so accelerates its degradation and distorts the ultrasound image.

While the phantom was designed to teach the FI block, other blocks with shared anatomy such as the femoral nerve and saphenous nerve blocks could also be taught with this phantom. Several iterations of this phantom were trialed prior to use in this educational session. The phantom presented here uses balloons for blood vessels, but in prior versions, latex tubing was used instead. While the balloons were easier to work with (easier to fill with water and avoid air bubbles) compared to the latex tubing, they did not prove as durable. Additionally, other nonperishable base medians were trialed including silicone and wax paraffin. Ballistics gel gave the best ultrasound images in our opinion. Using ballistics gel as a base also provides the opportunity to break the phantom down and reuse the gel if the model becomes overused or degraded. Theoretically, the same phantom could be made several times using the same original components by remelting the ballistics gel and thus increasing cost-effectiveness over time.

The phantom effectively taught EM residents and EM bound medical students proper needle placement for an FI block since all participants were able to demonstrate appropriate needle placement. The phantom was low cost, particularly compared to commercial trainers, and held up to a large number of needle sticks.

### Associated content

- Fascia Iliaca Block PowerPoint presentation

## Supplementary Information



## Figures and Tables

**Figure 1 and 2 f1-jetem-11-2-i43:**
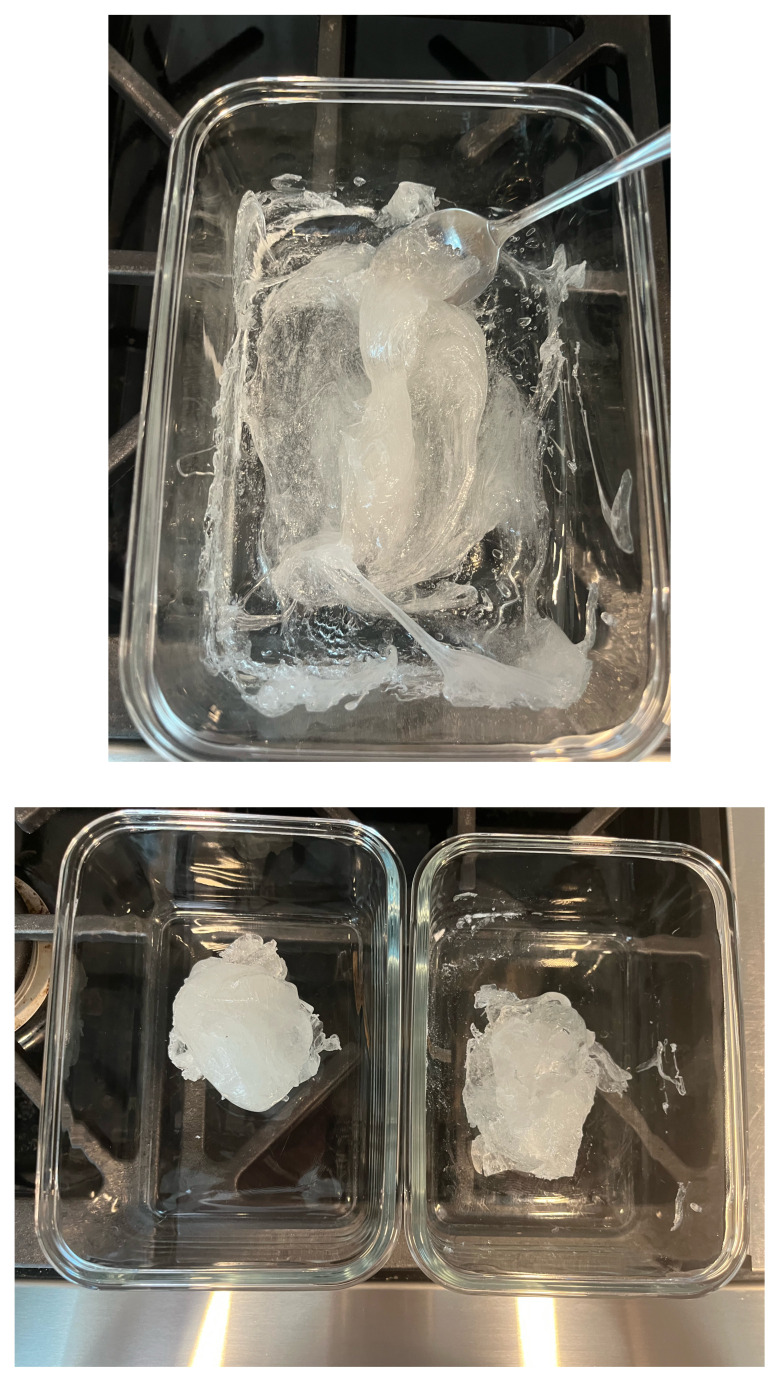
Mixing flour into the ballistics gel to create fascia: Author’s own image

**Figure 3 and 4 f2-jetem-11-2-i43:**
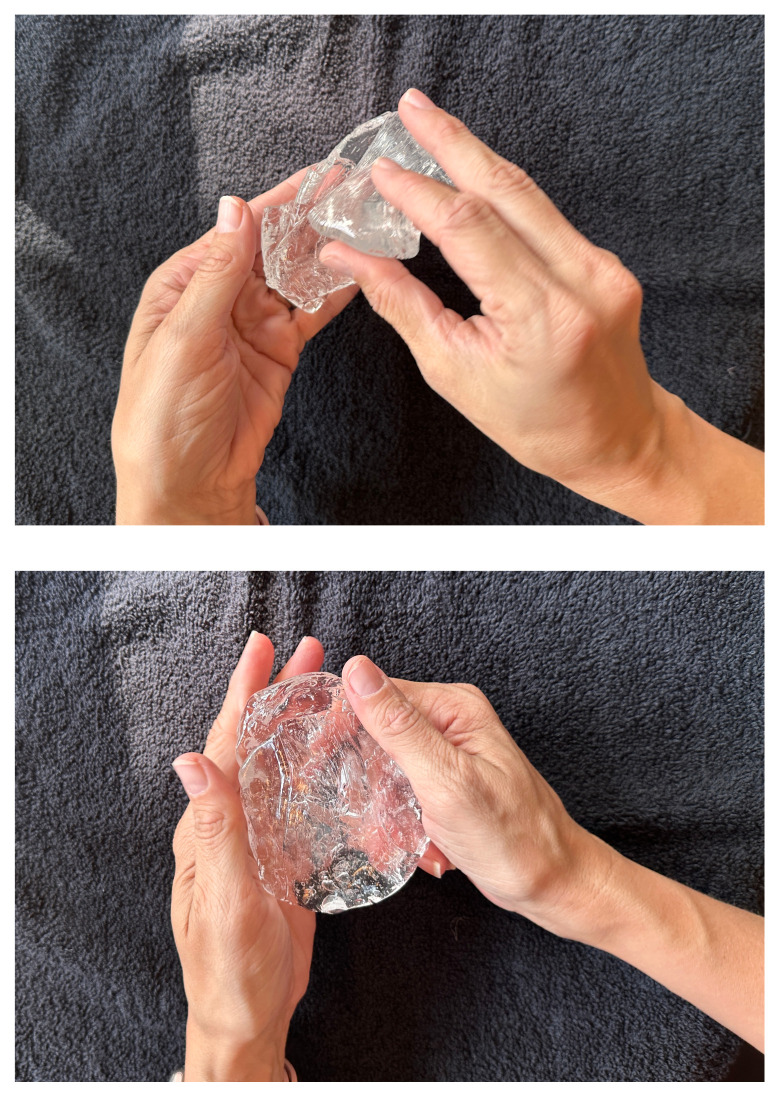
Molding ballistics gel to create muscle tissue: Author’s own images

**Figure 5 f3-jetem-11-2-i43:**
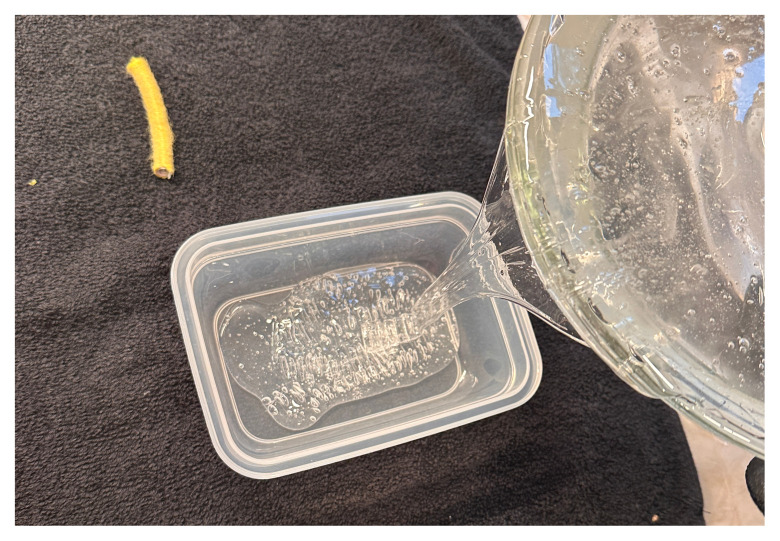
Pouring base layer of ballistics gel into phantom container: Author’s own image

**Figure 6 f4-jetem-11-2-i43:**
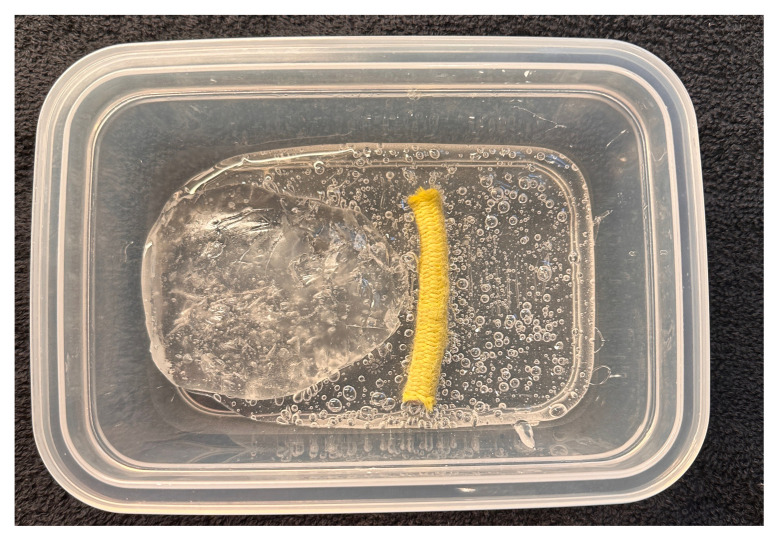
Placement of iliacus muscle and femoral nerve: Author’s own image

**Figure 7 f5-jetem-11-2-i43:**
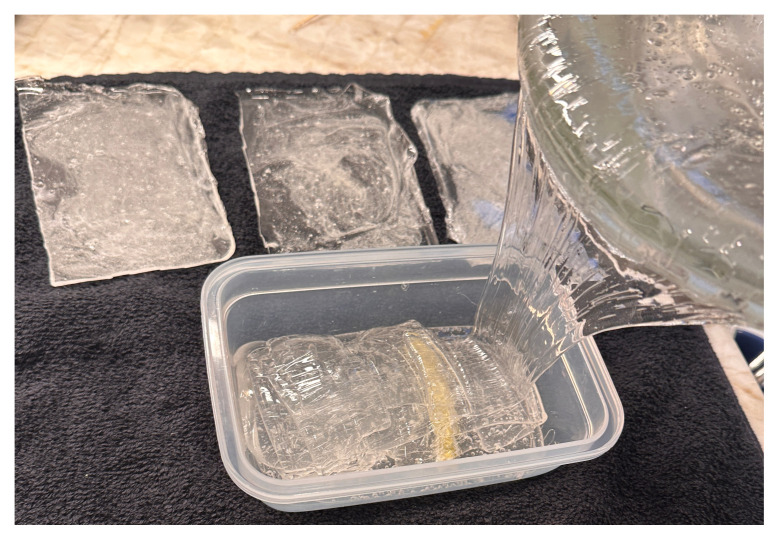
Pouring ballistics gel to cover Iliacus and femoral nerve: Author’s own image

**Figure 8 f6-jetem-11-2-i43:**
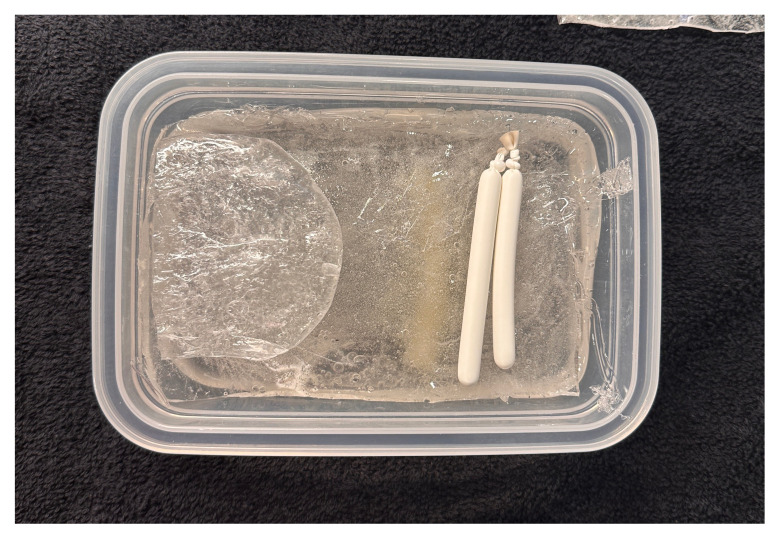
Placement of the sartorius muscle and femoral vessels on top of the fascia iliaca: Author’s own image

**Figure 9 and 10 f7-jetem-11-2-i43:**
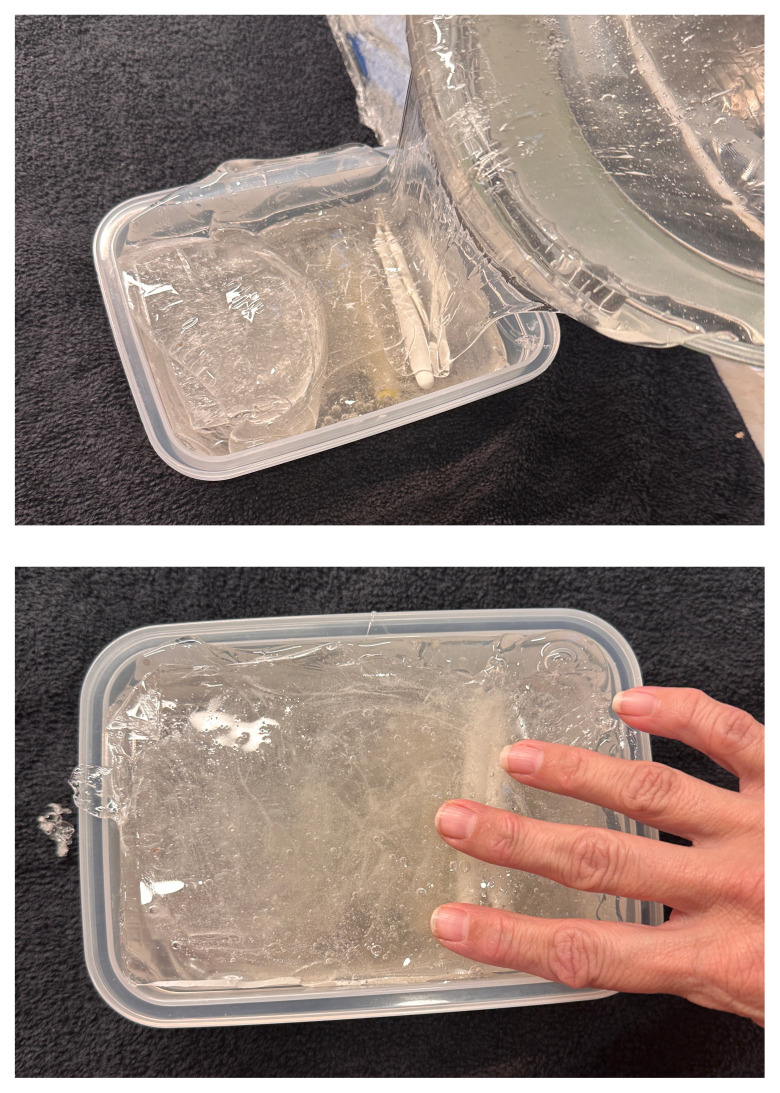
Pouring ballistics gel over the sartorius and femoral vessels and placing fascia lata on top: Author’s own image

**Figure 11 f8-jetem-11-2-i43:**
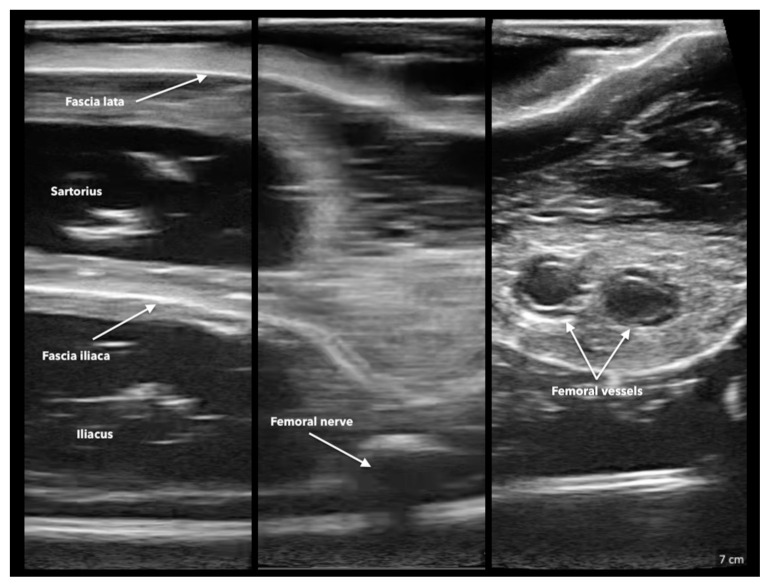
Phantom appearance on ultrasound: Author’s own image

**Figure:**
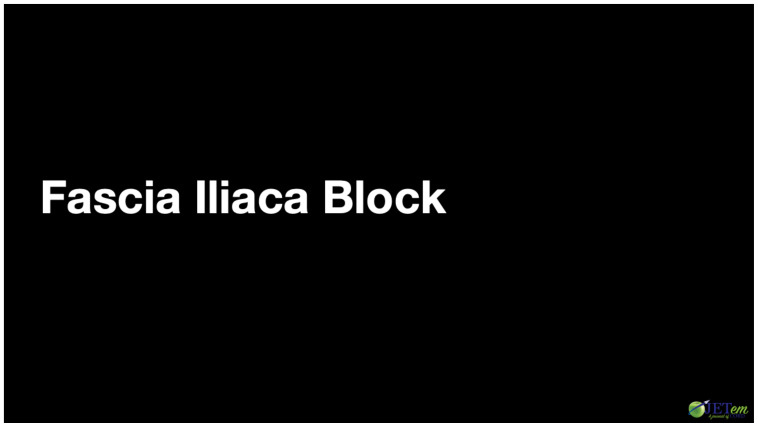
Please see associated PowerPoint file

## References

[1] Jain N, Kotulski C, Al-Hilli A, Yeung-Lai-Wah P, Pluta J, Heegeman D (2022). Fascia iliaca block in hip and femur fractures to reduce opioid use. J Emerg Med.

[2] Morrison S, Dickman E, Hwang U (2016). Regional nerve blocks improve pain and functional outcomes in hip fracture: A randomized controlled trial. J Am Geriatr Soc.

[3] Simulab Corporation Regional anesthesia femoral trainer with SmarTissue.

[4] Elevate Healthcare Gen II femoral vascular access & regional anesthesia ultrasound training model, hand pump.

[5] Rometti M, Keifer A, Wei G, Bryczkowski C (2024). Ultrasound-guided fascia iliaca nerve block gelatin model. JEM Reports.

[6] Parker C, Foster T (2020). Realistic tofu-based model for ultrasound-guided femoral nerve blocks. Vis J Emerg Med.

[7] Micheller D, Chapman M, Cover M (2017). A low-fidelity, high-functionality, inexpensive ultrasound-guided nerve block model. CJEM.

[8] Vyas AJ, Lo MC, AU A A konnyaku jelly model for ultrasound-guided fascia iliaca compartment block. 2024. Alpha Omega Alpha Research Symposium Posters.

[9] Sparks S, Evans D, Byars D (2014). A low-cost, high-fidelity nerve block model. Crit Ultrasound J.

[10] Naraghi L, Lin J, Odashima K, Buttar S, Haines L, Dickman E (2019). Ultrasound-guided regional anesthesia simulation: Use of meat glue in inexpensive and realistic nerve block models. BMC Med Educ.

[11] Malik A, Thom S, Haber B (2022). Regional anesthesia in the emergency department: An overview of common nerve block techniques and recent literature. Curr Emerg Hosp Med Rep.

[12] Ketelaars R, Stollman J, van Eeten E, Eikendal T, Bruhn J, van Geffen G (2018). Emergency physician-performed ultrasound-guided nerve blocks in proximal femoral fractures provide safe and effective pain relief: A prospective observational study in the Netherlands. Int J Emerg Med.

[13] Proposed Revisions Emergency Medicine Defined Key Index Procedure Minimums Review Committee for Emergency Medicine ACGME.

[14] Council of Residency Directors in Emergency Medicine Model curriculum Appendix C emergency medicine goals and objectives.

