# Supplementary material for: A Novel Low-Cost Phantom for Ultrasound-Guided Fascia Iliaca Nerve Blocks
Source: J Educ Teach Emerg Med. 2026 Apr 30;11(2):I43–50. doi: 10.5070/M5.52321 (PMC13152351; doi:10.5070/M5.52321)
Supplement: Supplementary file 1 [file 11-2-I43-Appendix_A.pptx]

## Slide 1
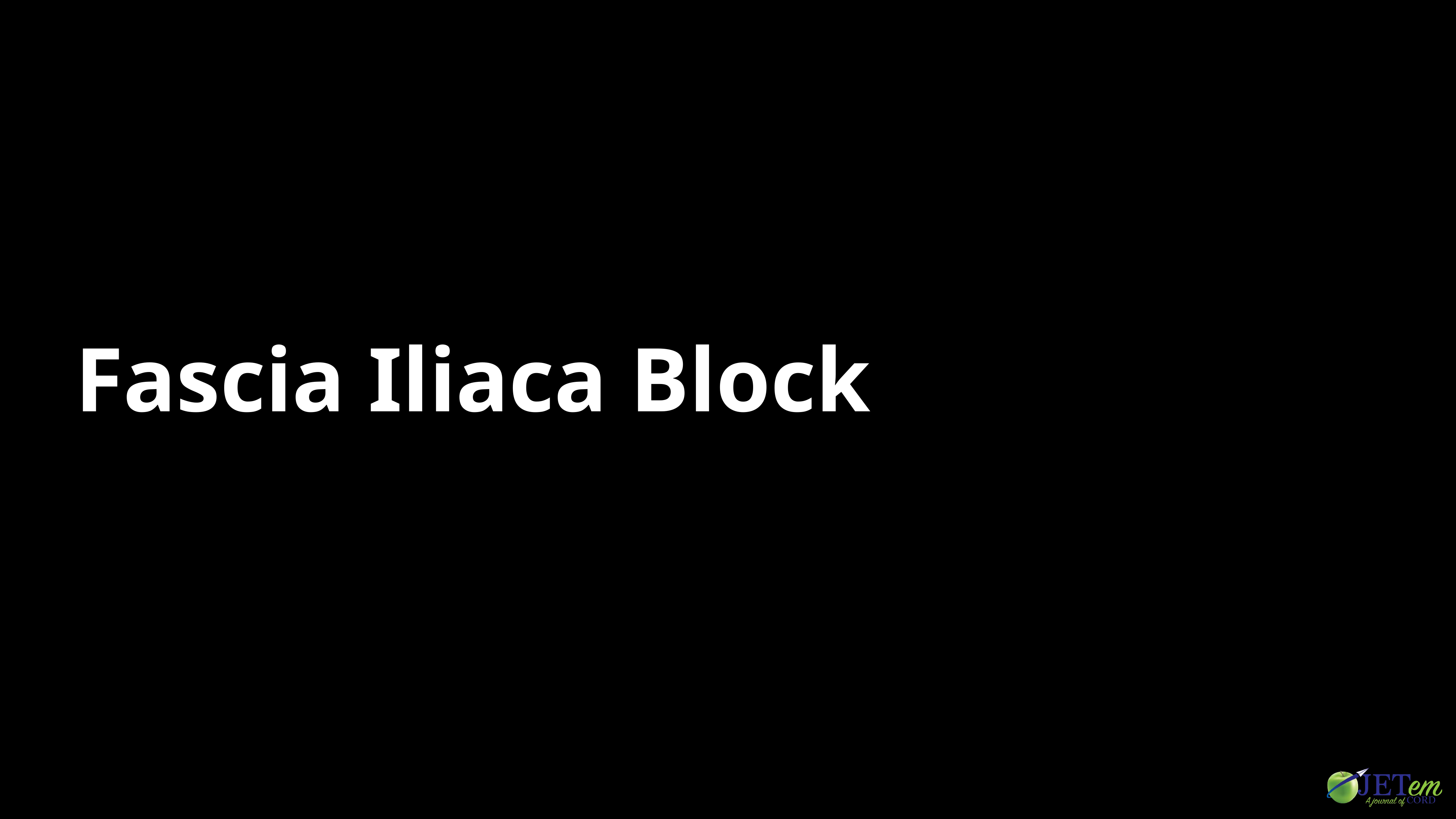

Fascia Iliaca Block

## Slide 2
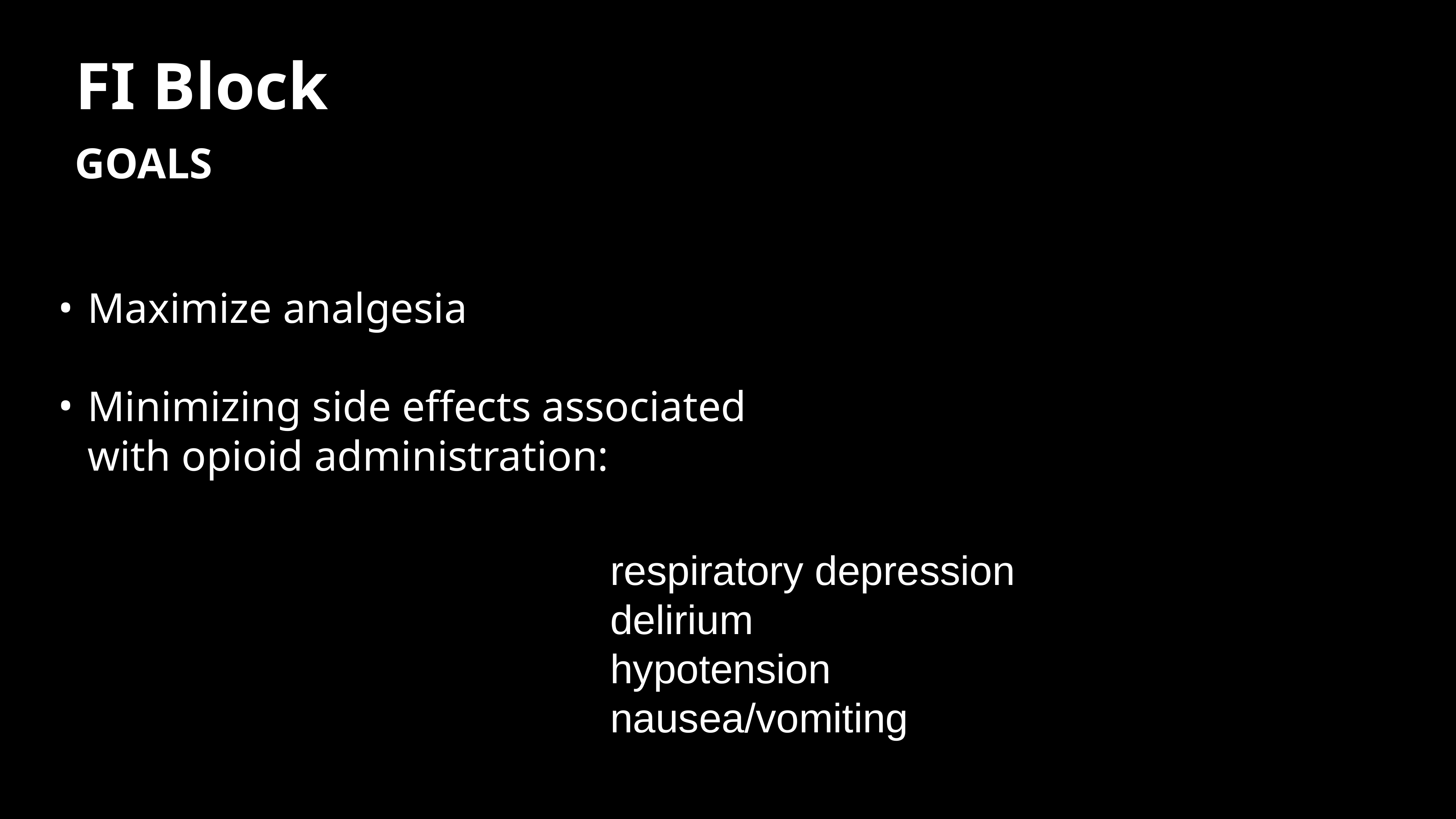

# FI Block
GOALS
Maximize analgesia
Minimizing side effects associated with opioid administration:
respiratory depression
delirium
hypotension
nausea/vomiting

## Slide 3
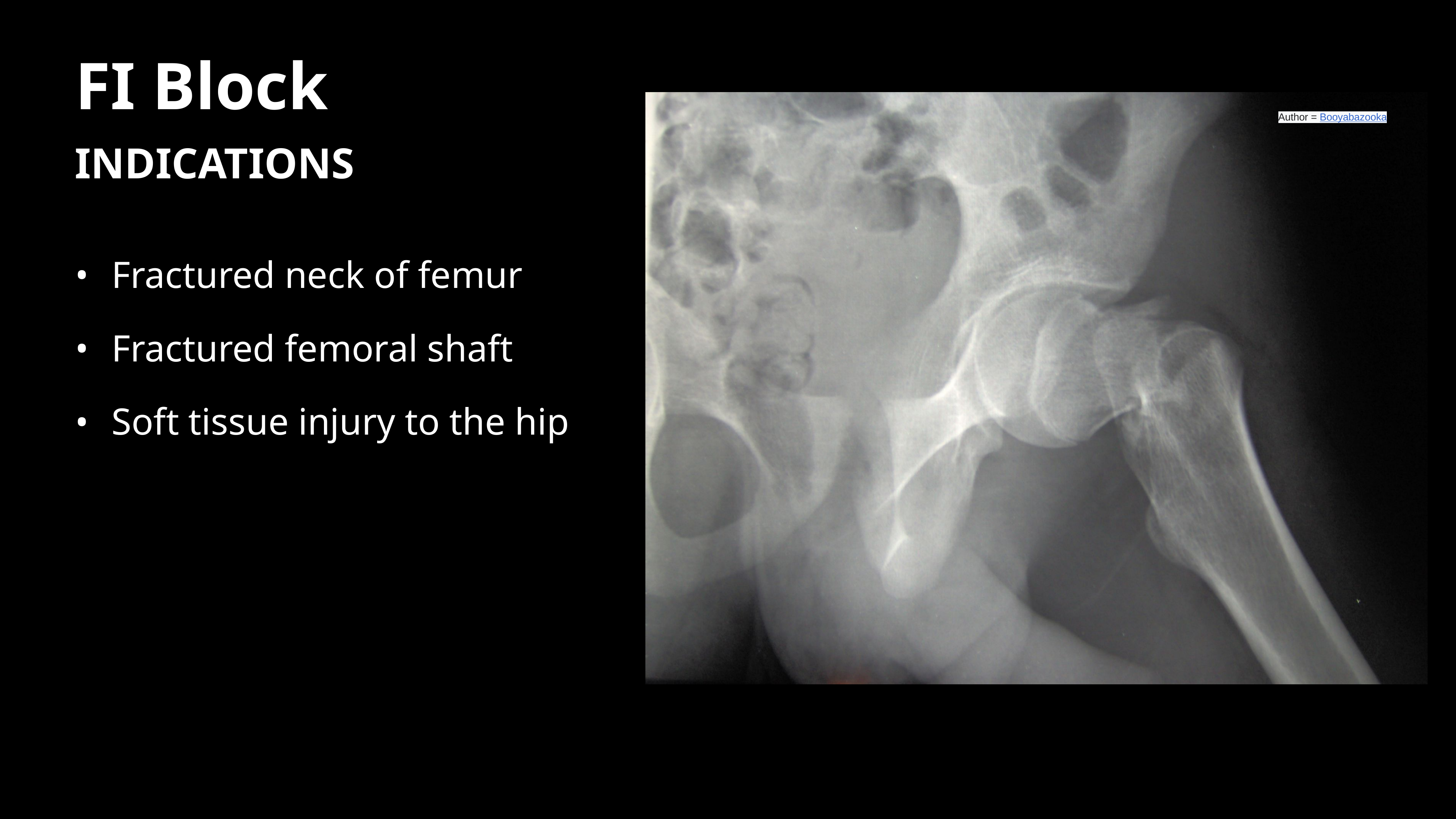

# FI Block
Author = Booyabazooka
INDICATIONS
Fractured neck of femur
Fractured femoral shaft
Soft tissue injury to the hip

## Slide 4
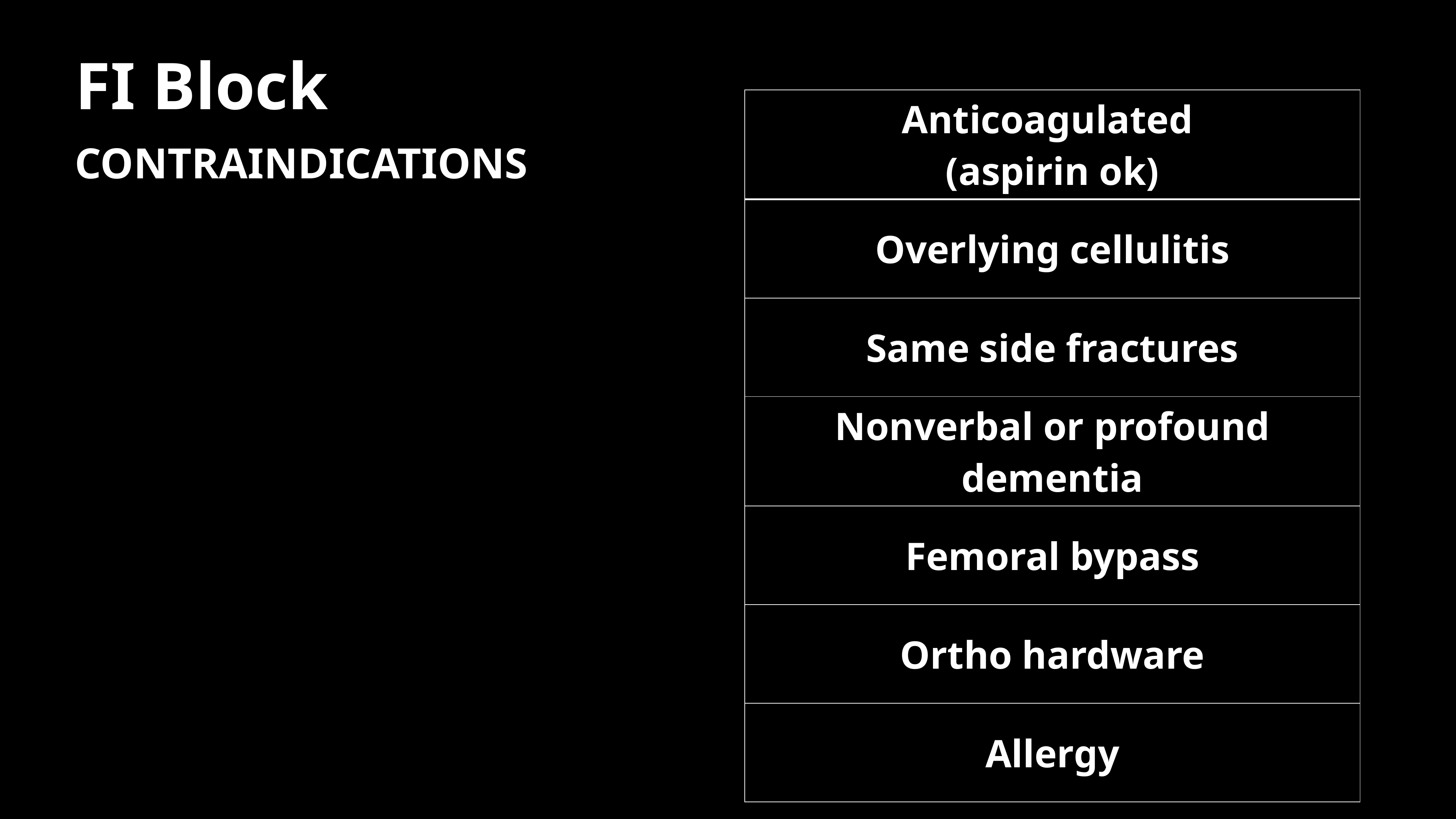

# FI Block
| Anticoagulated (aspirin ok) |
| --- |
| Overlying cellulitis |
| Same side fractures |
| Nonverbal or profound dementia |
| Femoral bypass |
| Ortho hardware |
| Allergy |
CONTRAINDICATIONS

## Slide 5
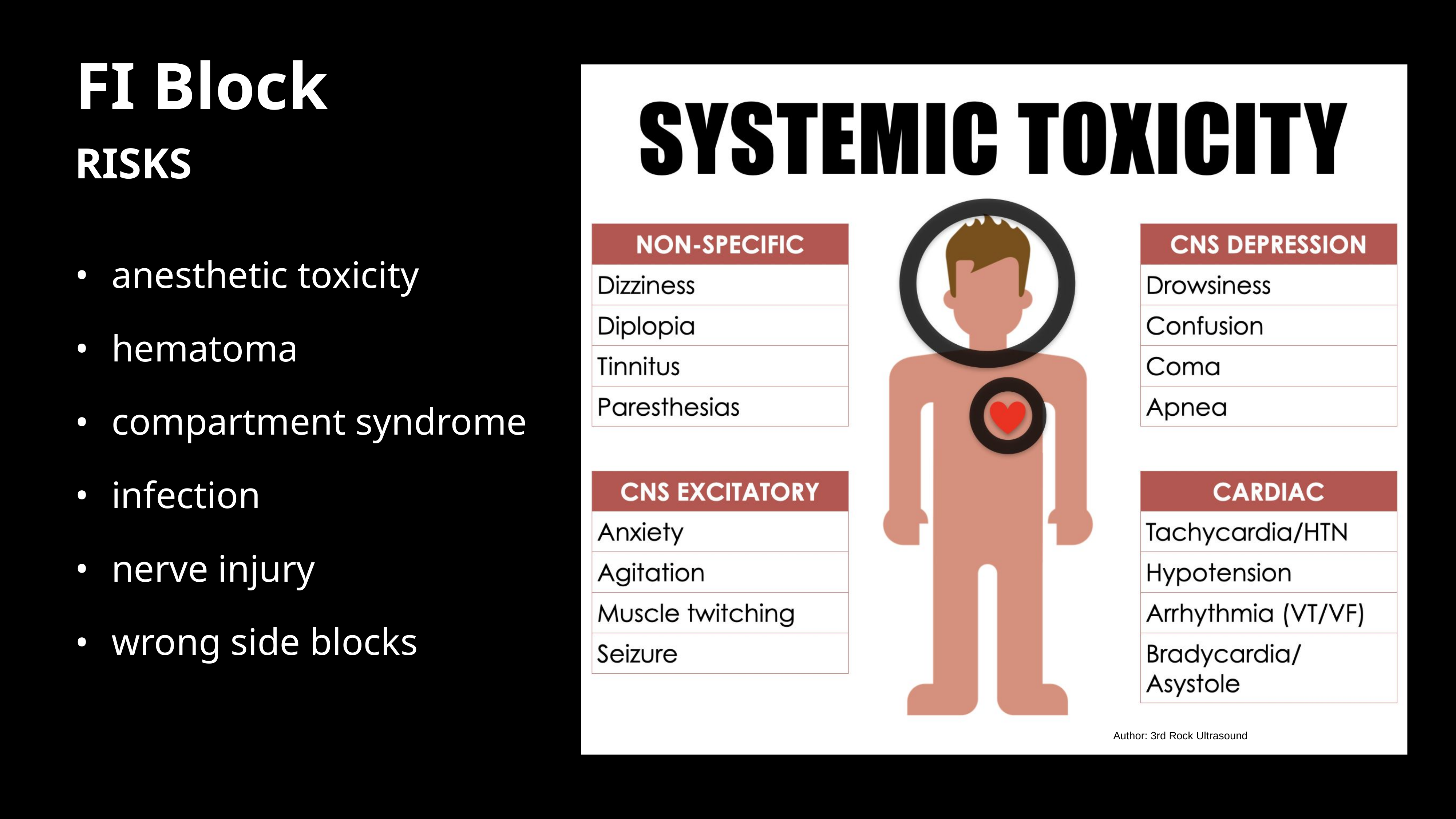

# FI Block
RISKS
anesthetic toxicity
hematoma
compartment syndrome
infection
nerve injury
wrong side blocks
Author: 3rd Rock Ultrasound

## Slide 6
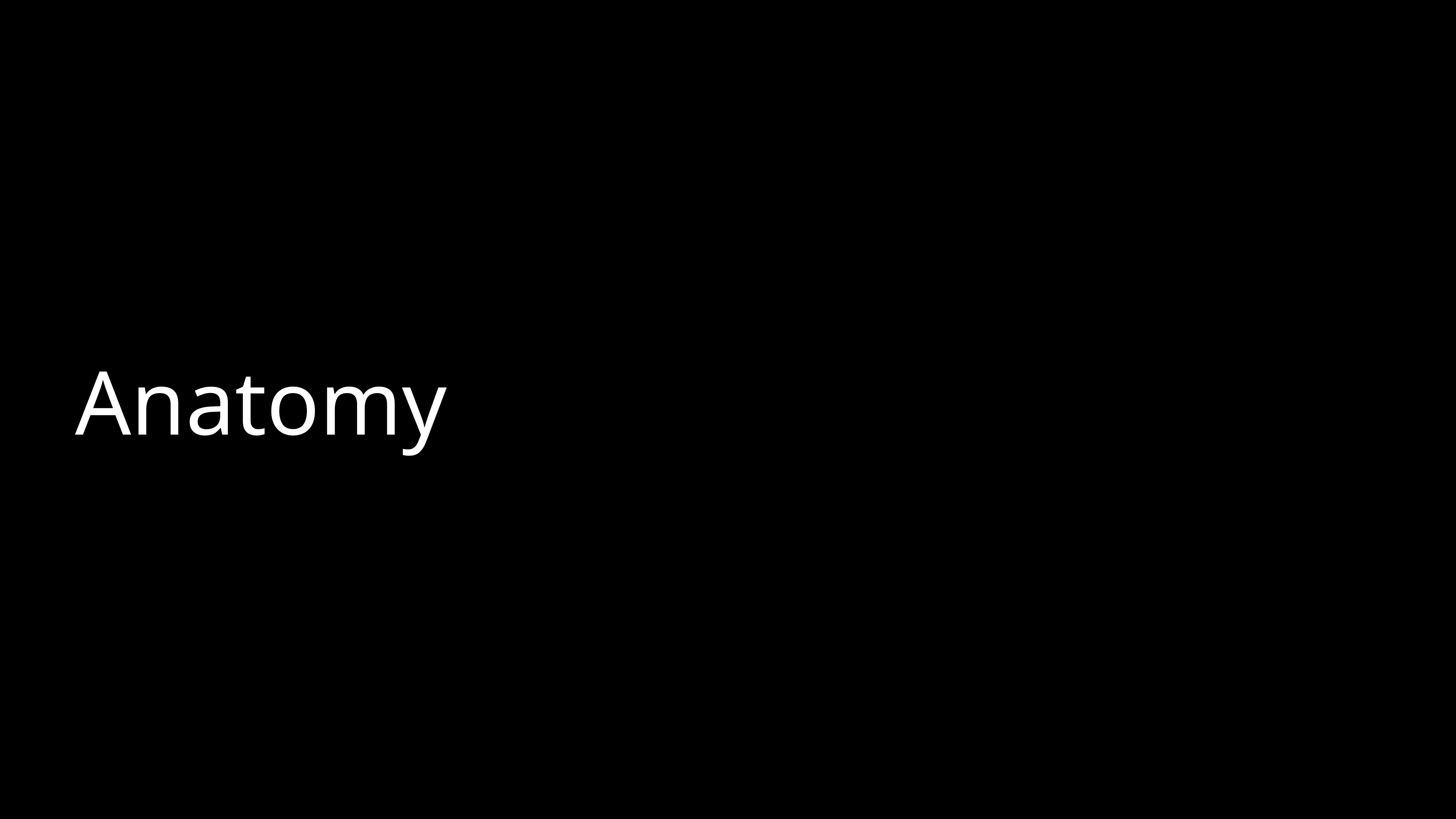

# Anatomy

## Slide 7
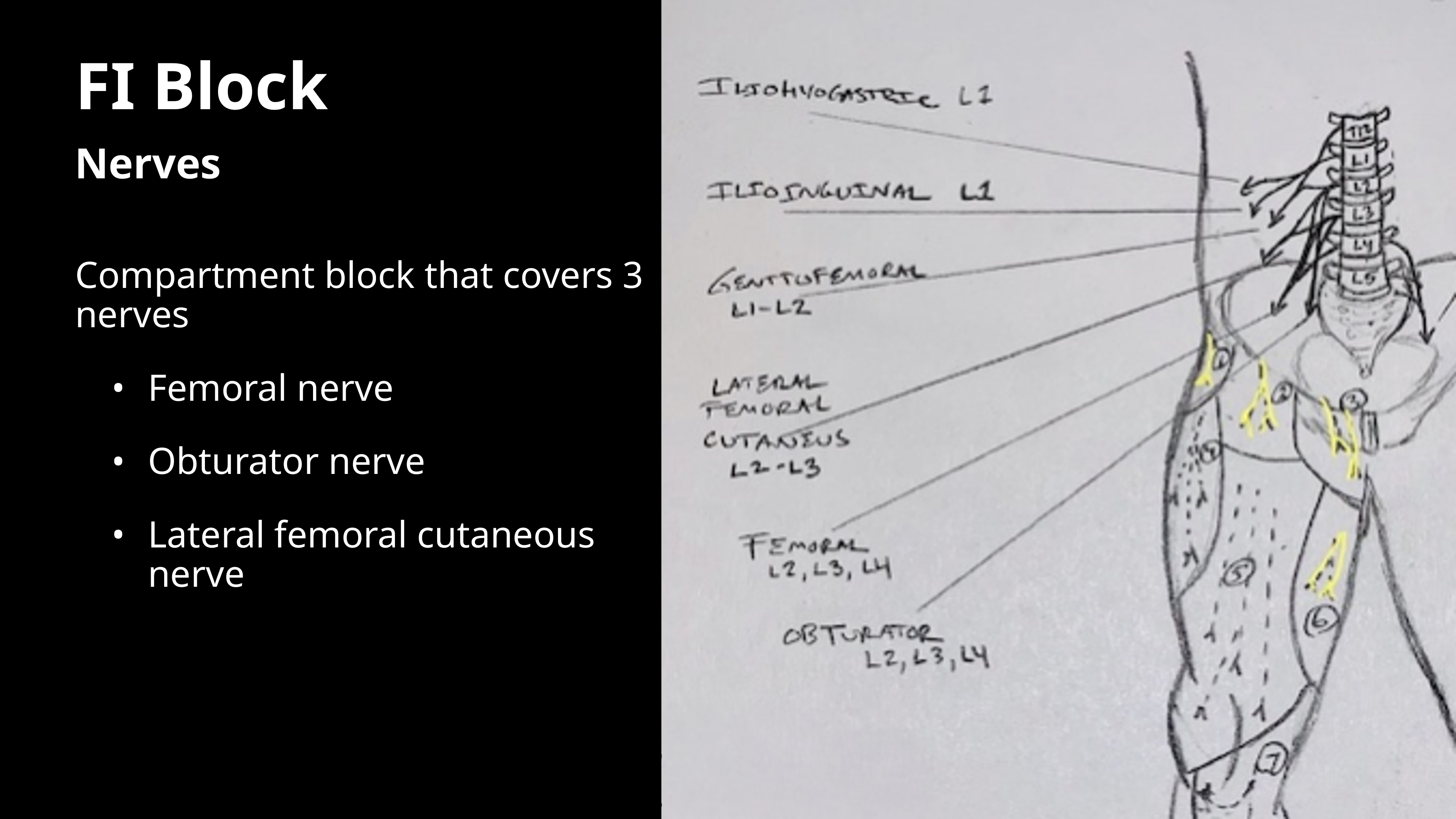

# FI Block
Nerves
Compartment block that covers 3 nerves
Femoral nerve
Obturator nerve
Lateral femoral cutaneous nerve

## Slide 8
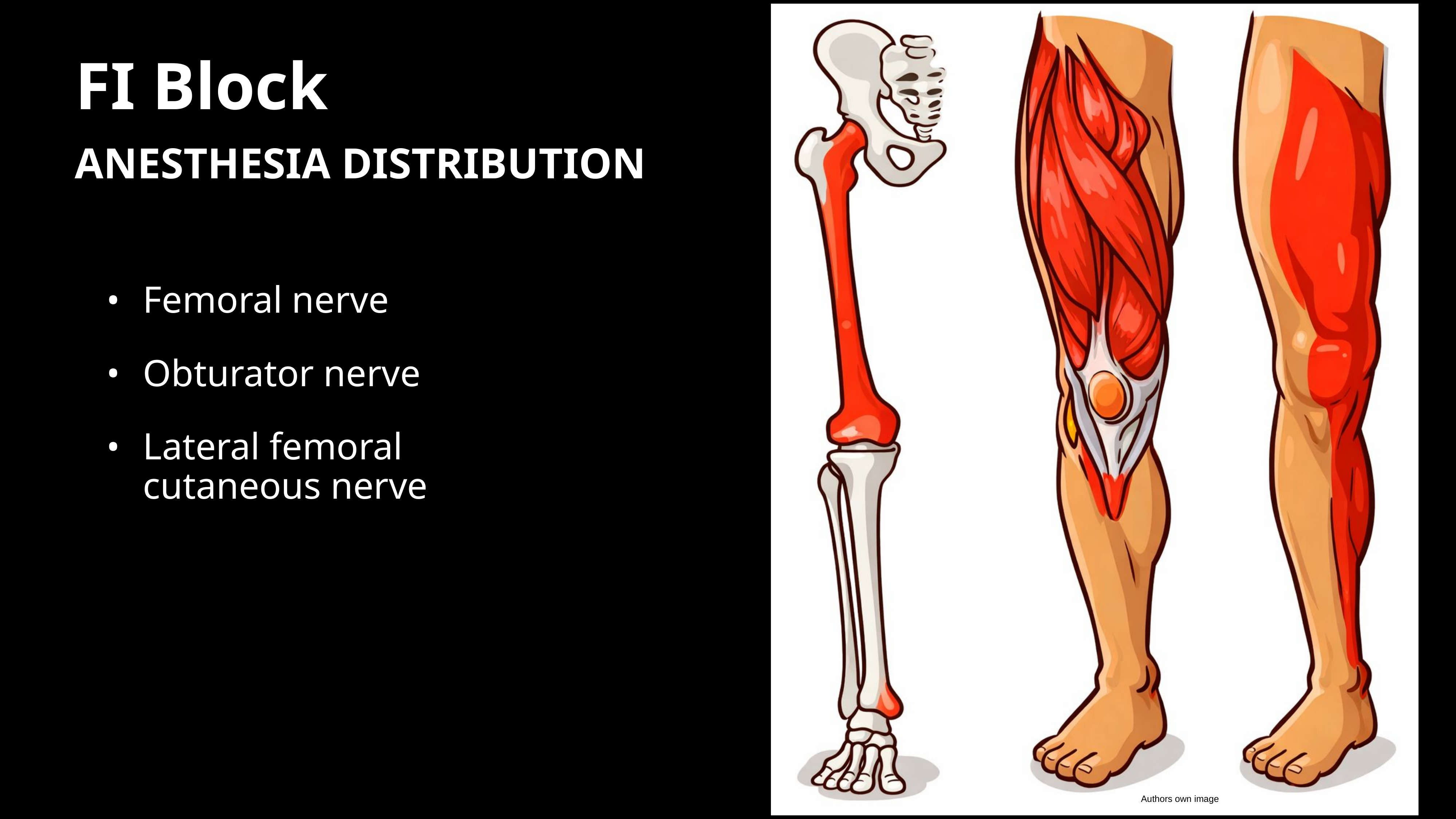

FI Block
ANESTHESIA DISTRIBUTION
Femoral nerve
Obturator nerve
Lateral femoral cutaneous nerve
Authors own image

## Slide 9
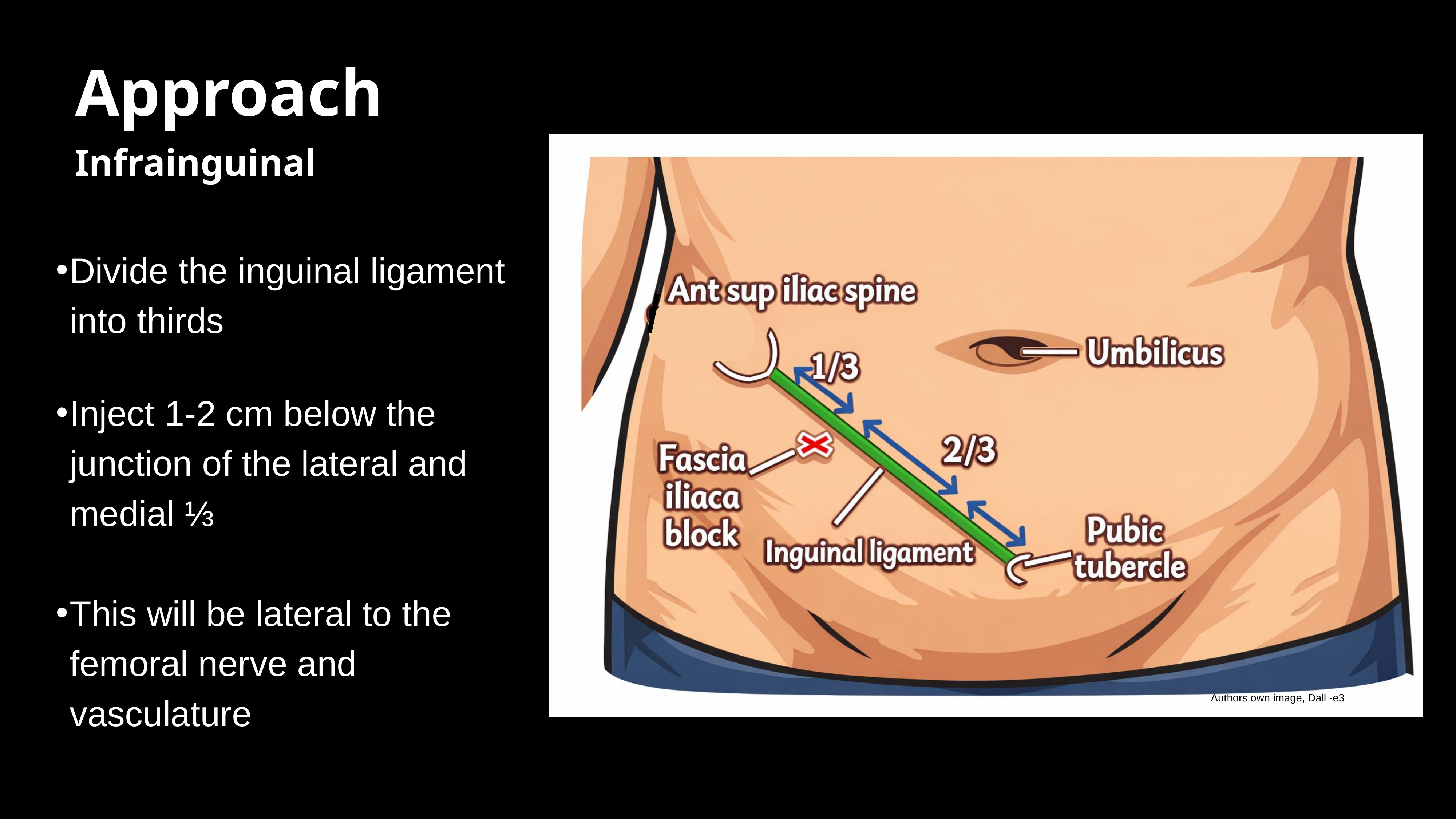

# Approach
Infrainguinal
Divide the inguinal ligament into thirds
Inject 1-2 cm below the junction of the lateral and medial ⅓
This will be lateral to the femoral nerve and vasculature
Authors own image, Dall -e3

## Slide 10
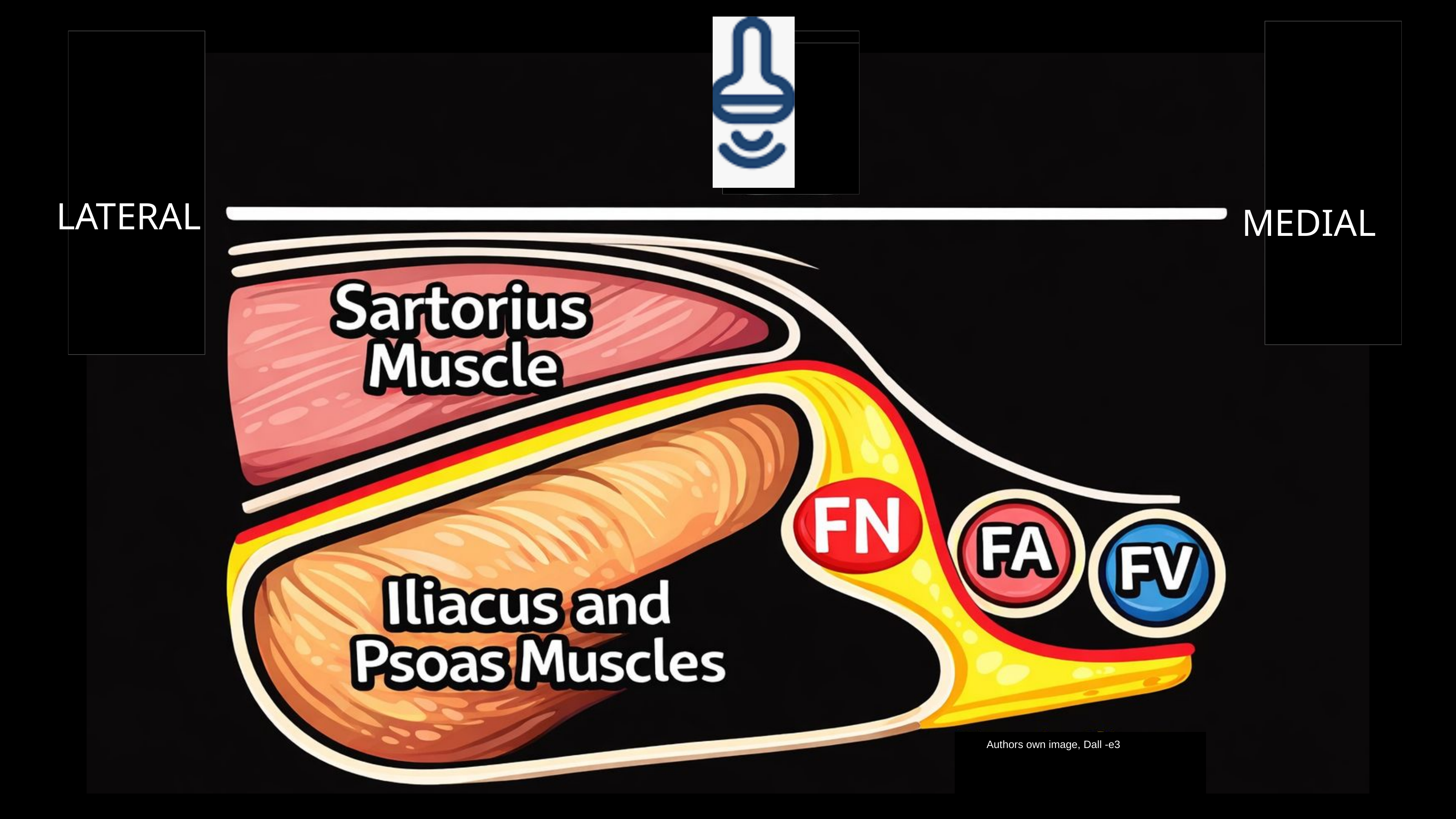

LATERAL
MEDIAL
Authors own image, Dall -e3

## Slide 11
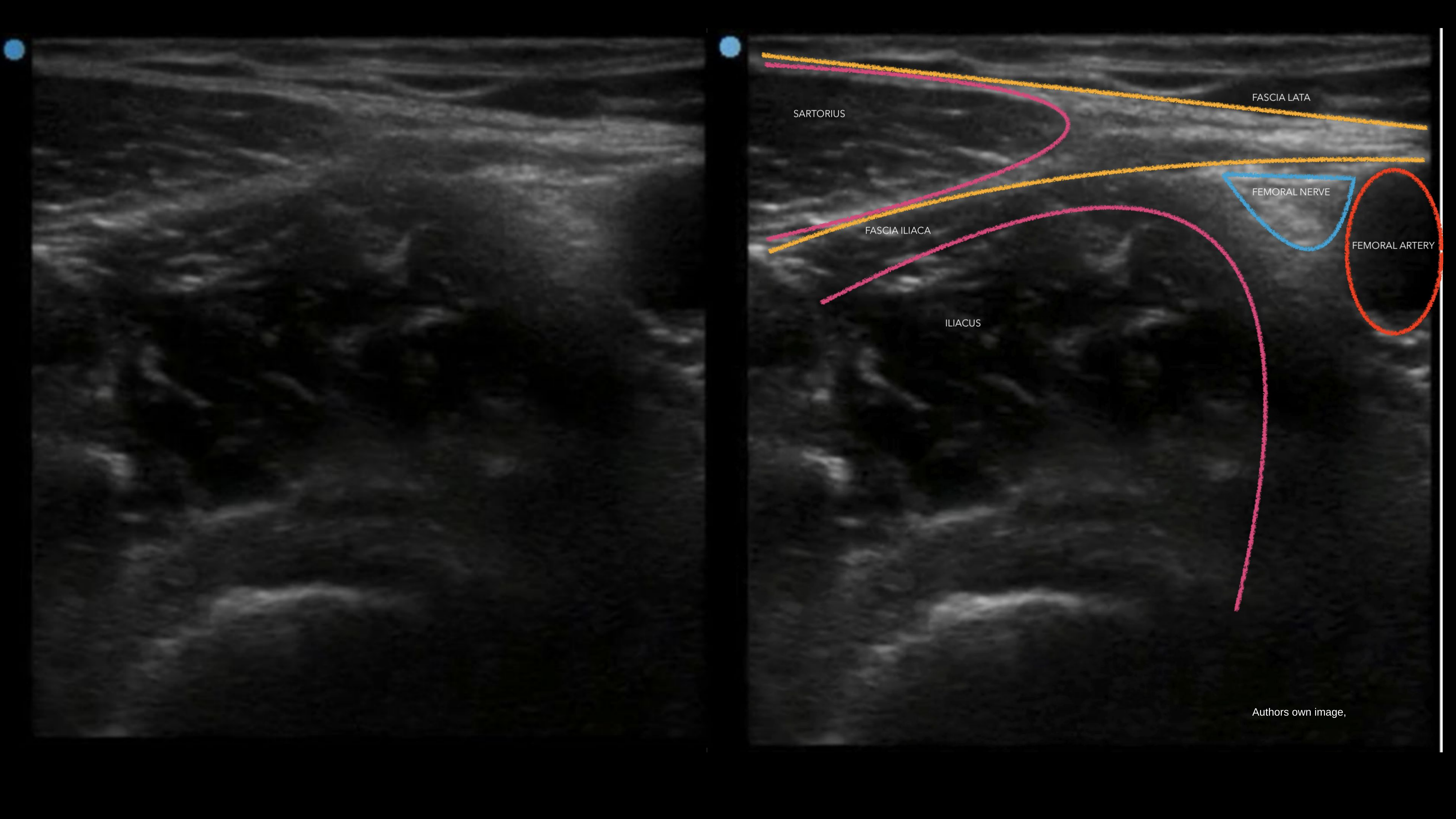

Authors own image,

## Slide 12
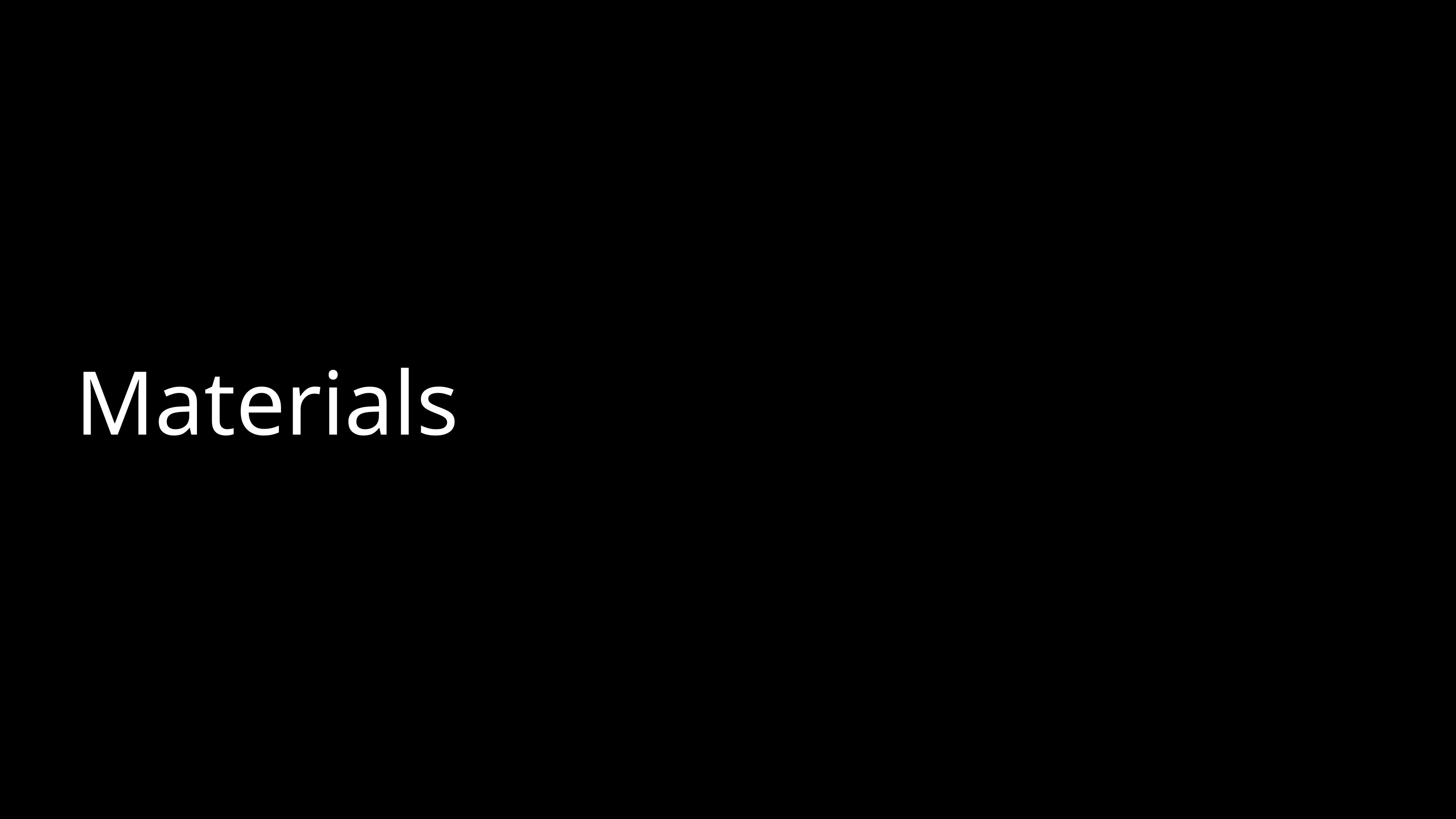

# Materials

## Slide 13
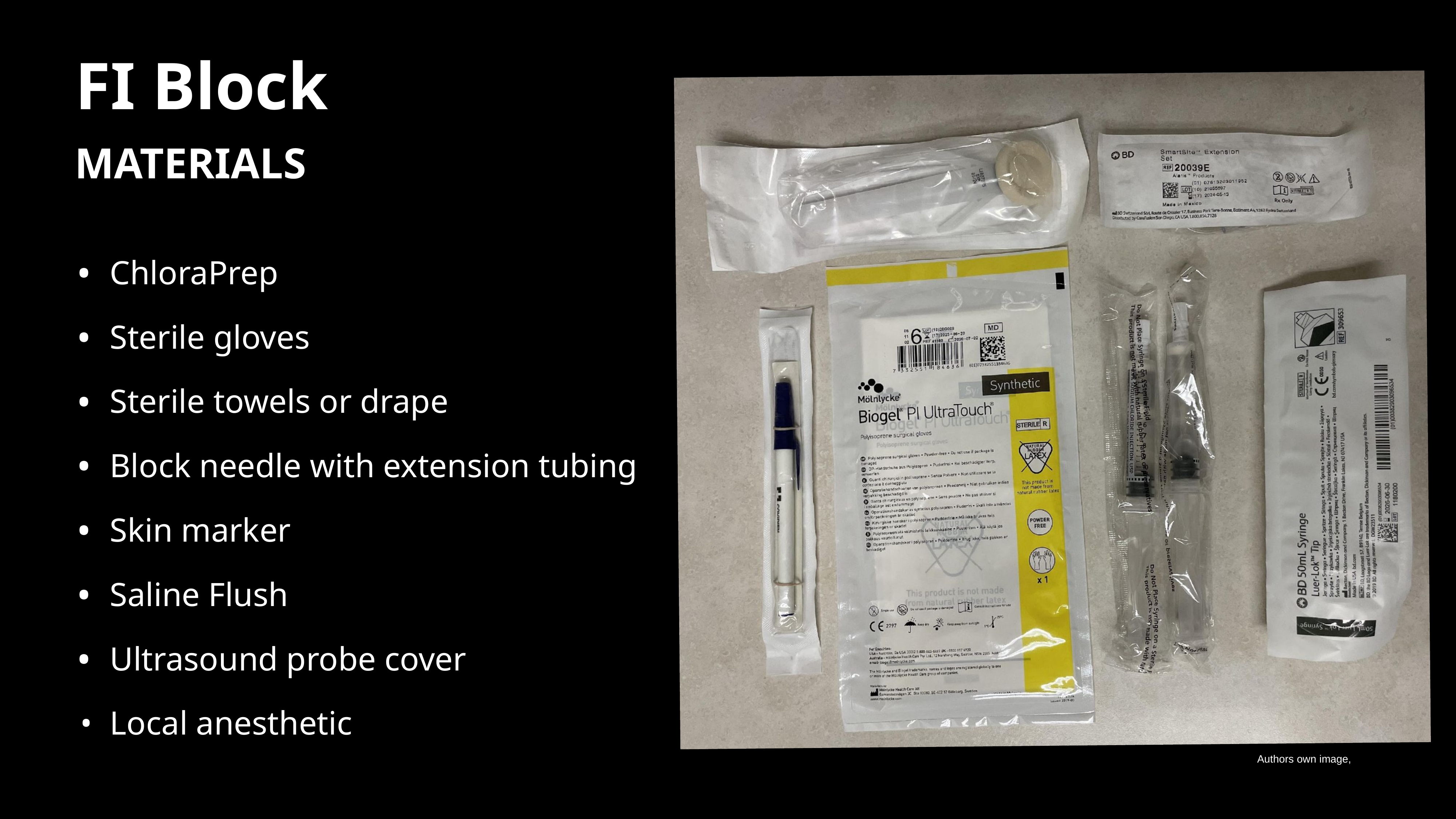

# FI Block
MATERIALS
ChloraPrep
Sterile gloves
Sterile towels or drape
Block needle with extension tubing
Skin marker
Saline Flush
Ultrasound probe cover
Local anesthetic
Authors own image,

## Slide 14
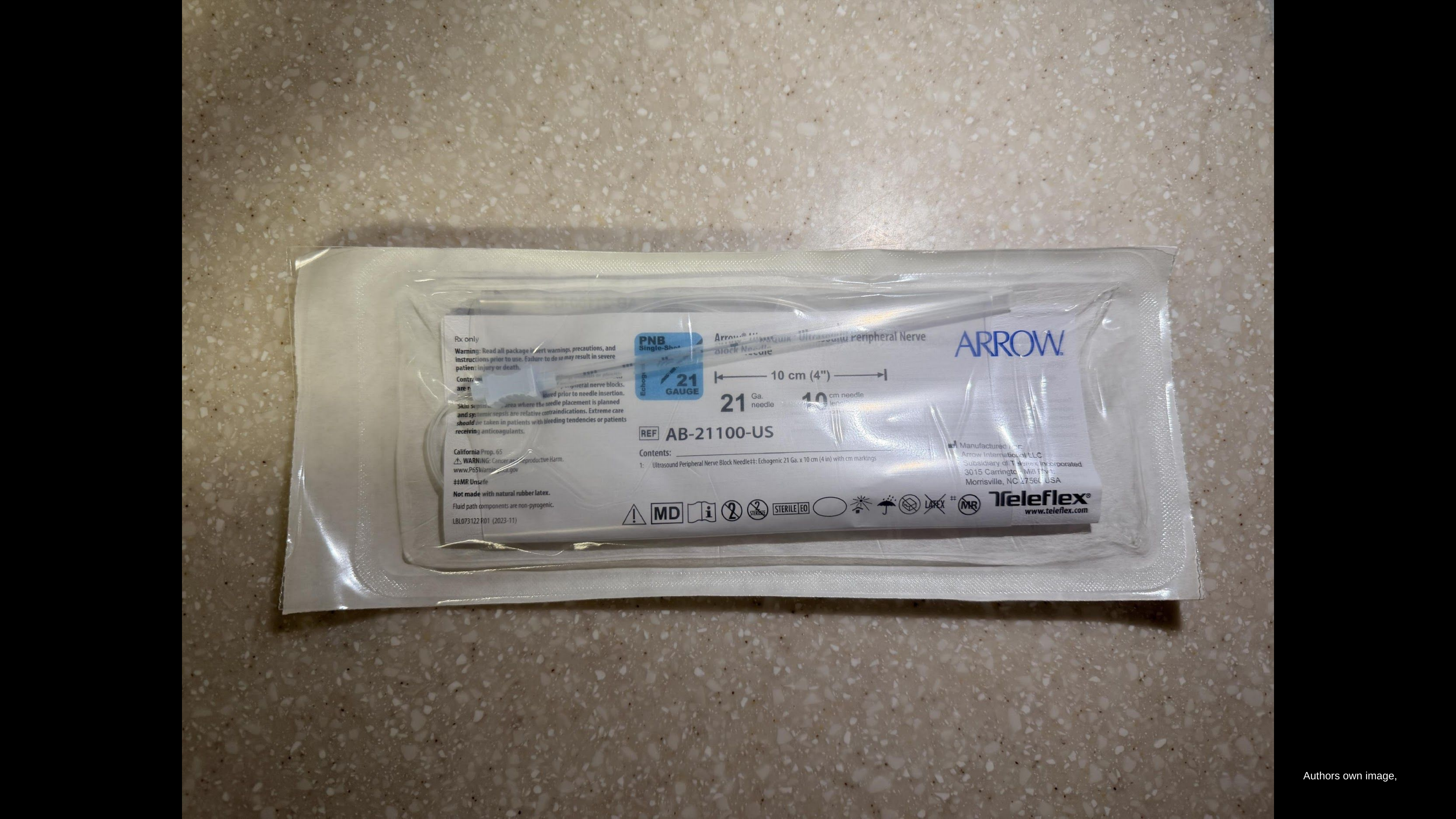

Authors own image,

## Slide 15
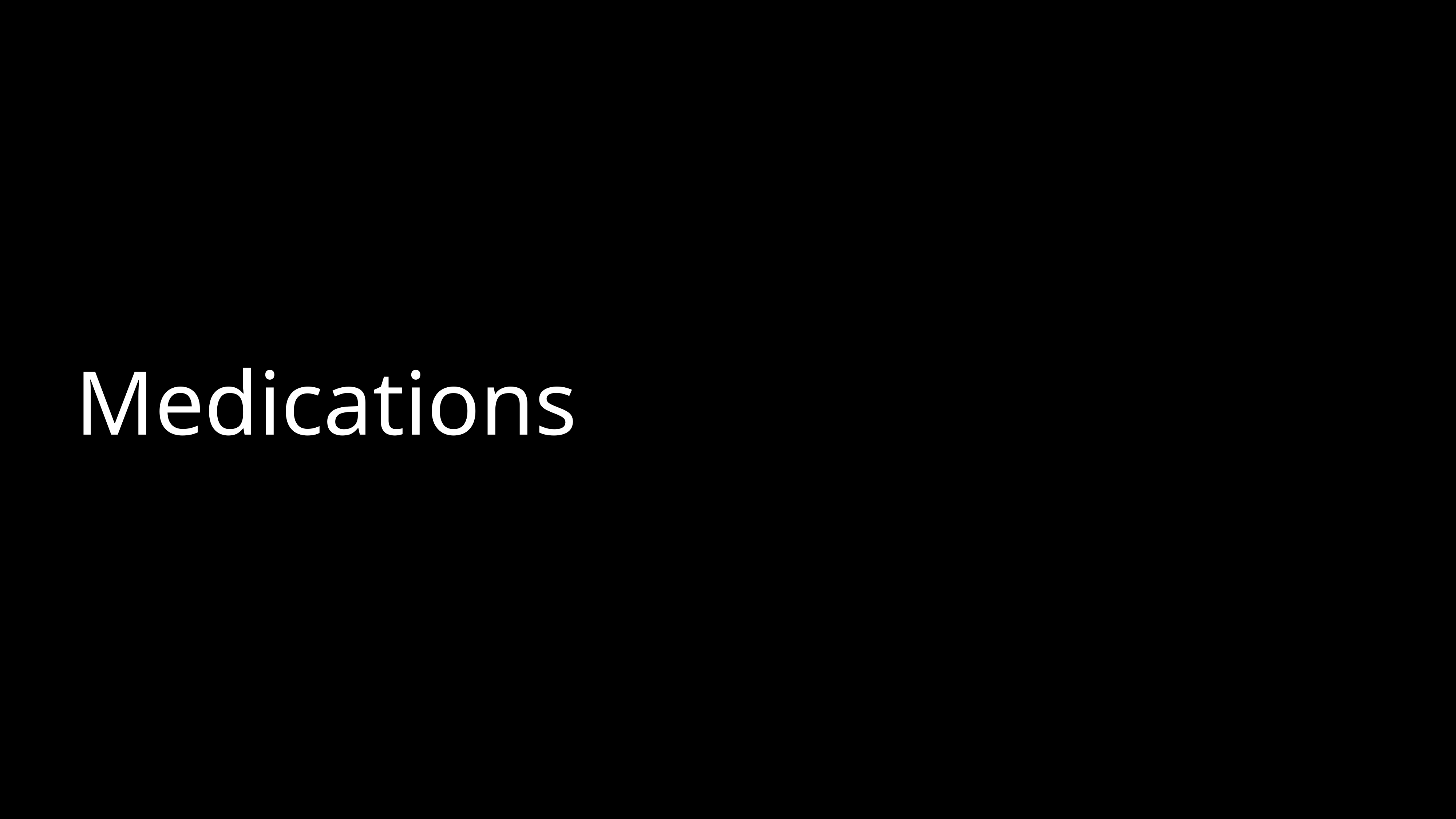

# Medications

## Slide 16
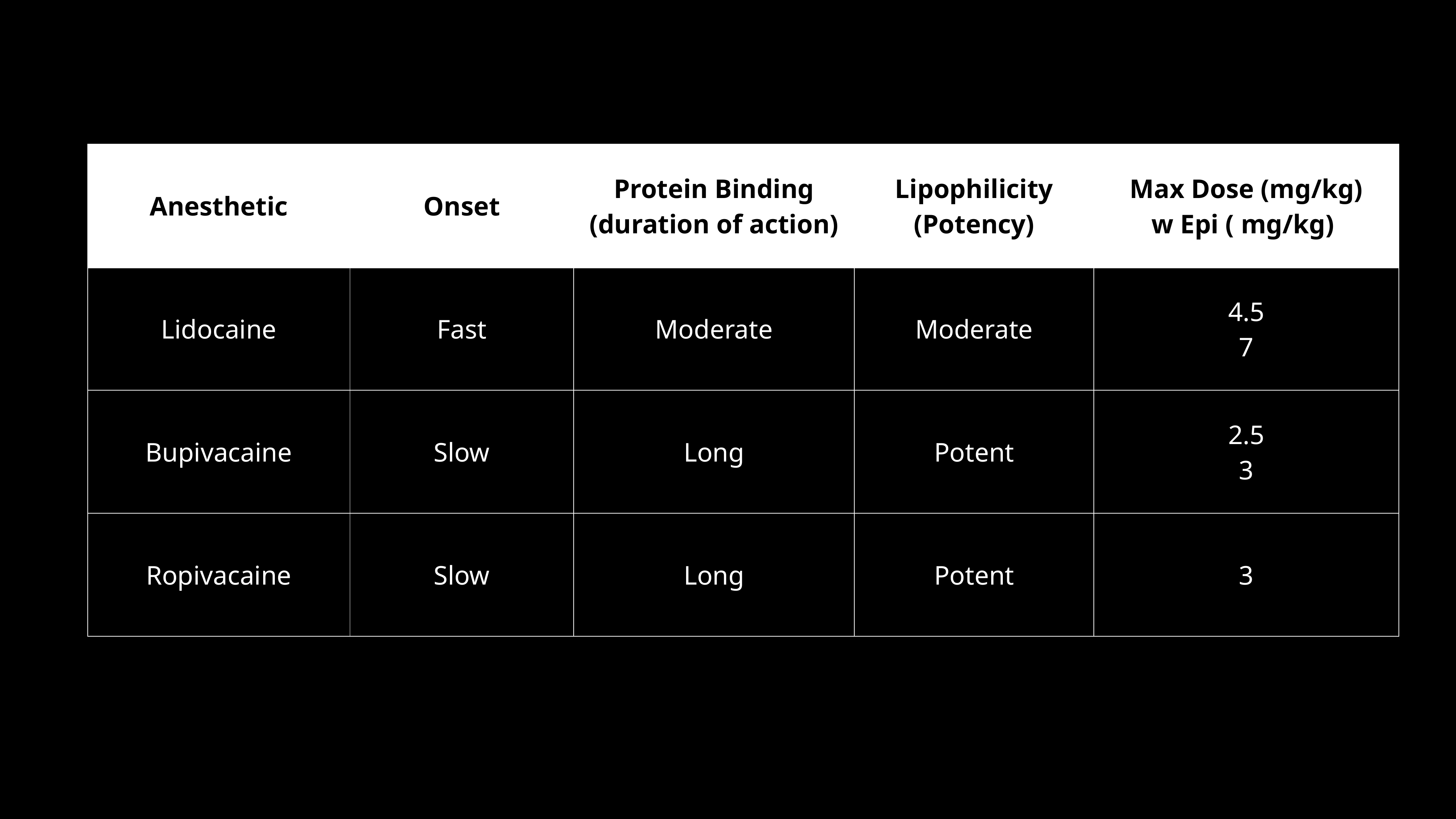

| Anesthetic | Onset | Protein Binding (duration of action) | Lipophilicity (Potency) | Max Dose (mg/kg) w Epi ( mg/kg) |
| --- | --- | --- | --- | --- |
| Lidocaine | Fast | Moderate | Moderate | 4.5 7 |
| Bupivacaine | Slow | Long | Potent | 2.5 3 |
| Ropivacaine | Slow | Long | Potent | 3 |

## Slide 17
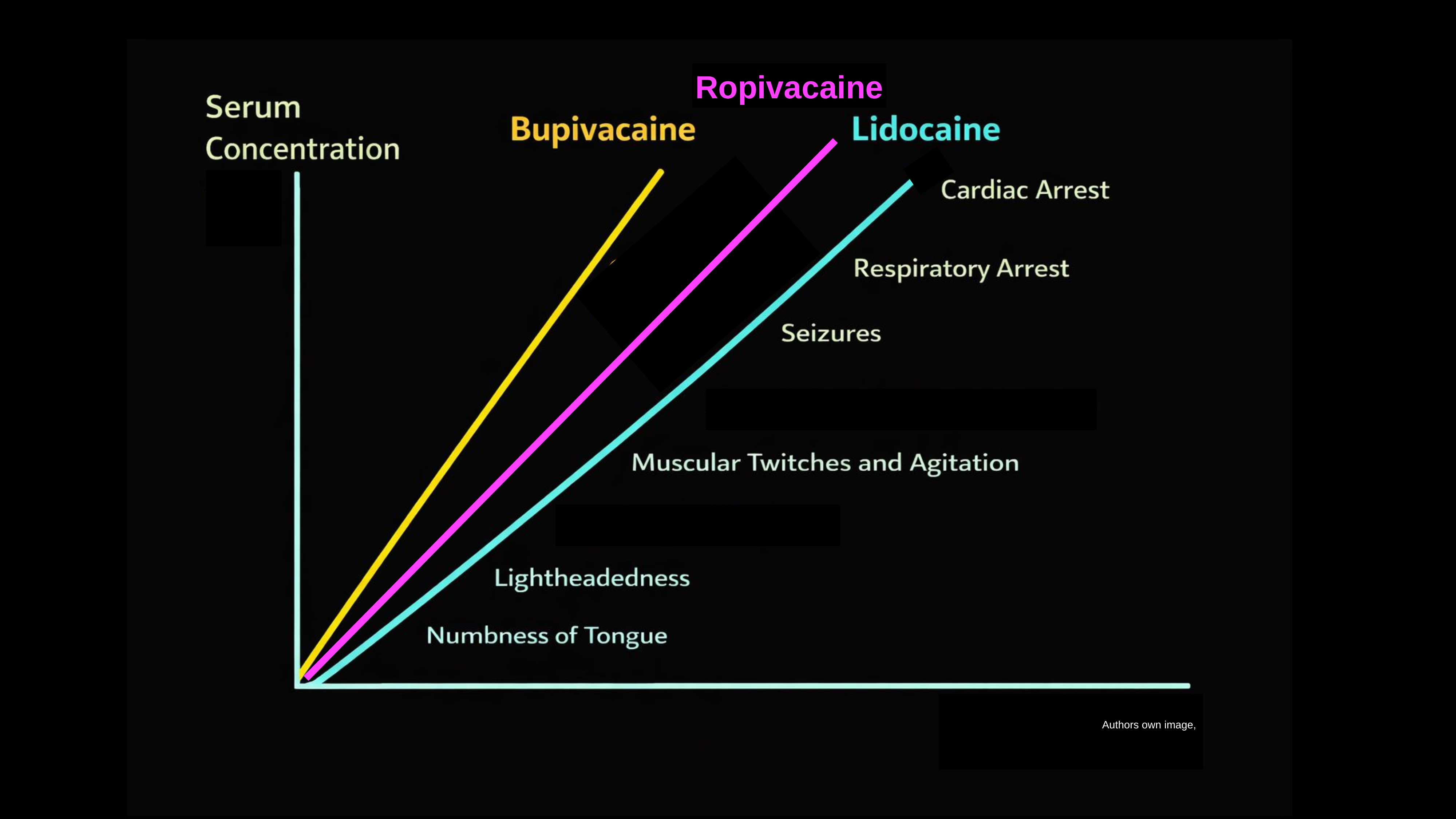

Ropivacaine
Authors own image,

## Slide 18
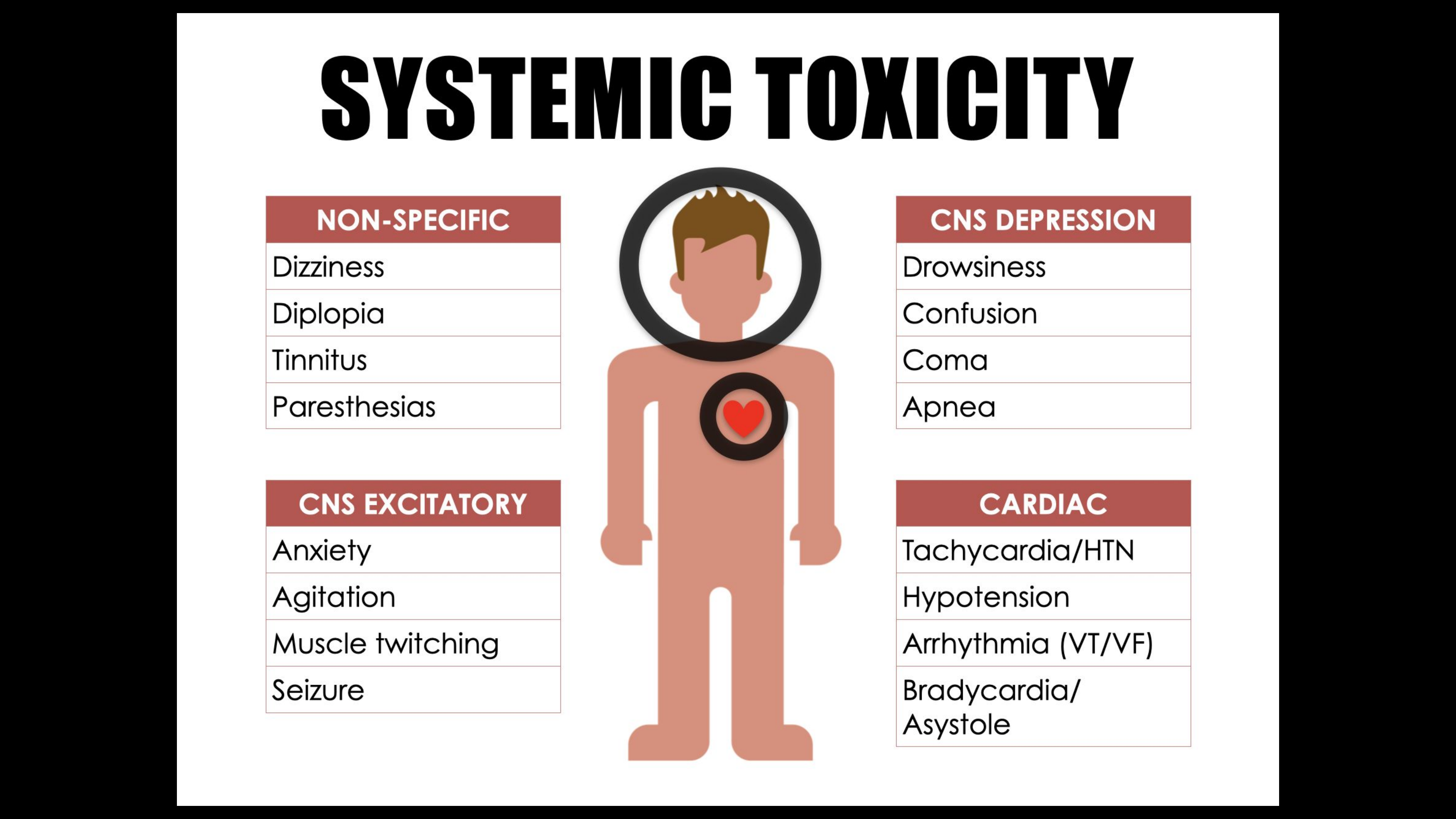

## Slide 19
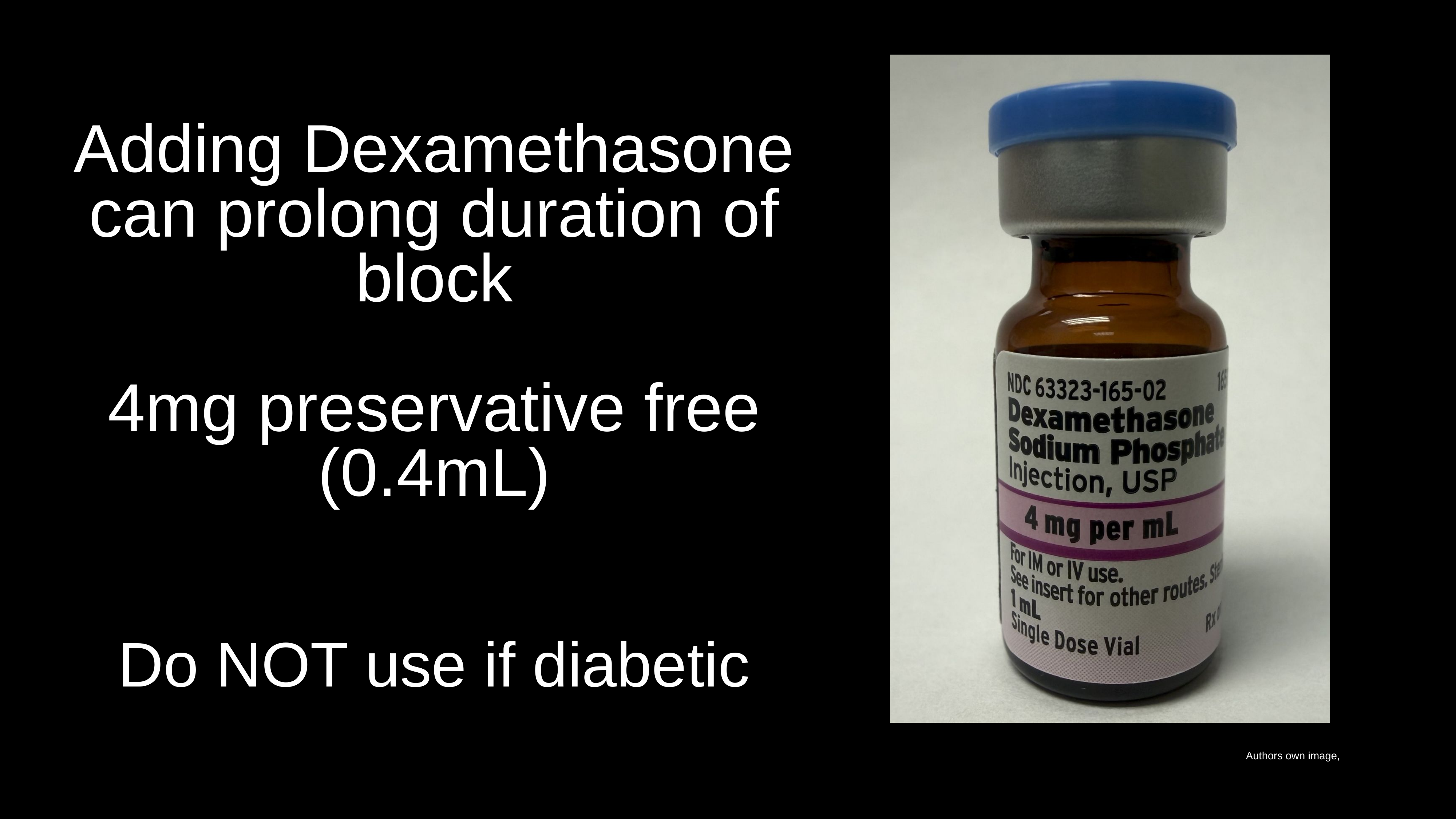

Adding Dexamethasone can prolong duration of block
4mg preservative free
(0.4mL)
Do NOT use if diabetic
Authors own image,

## Slide 20
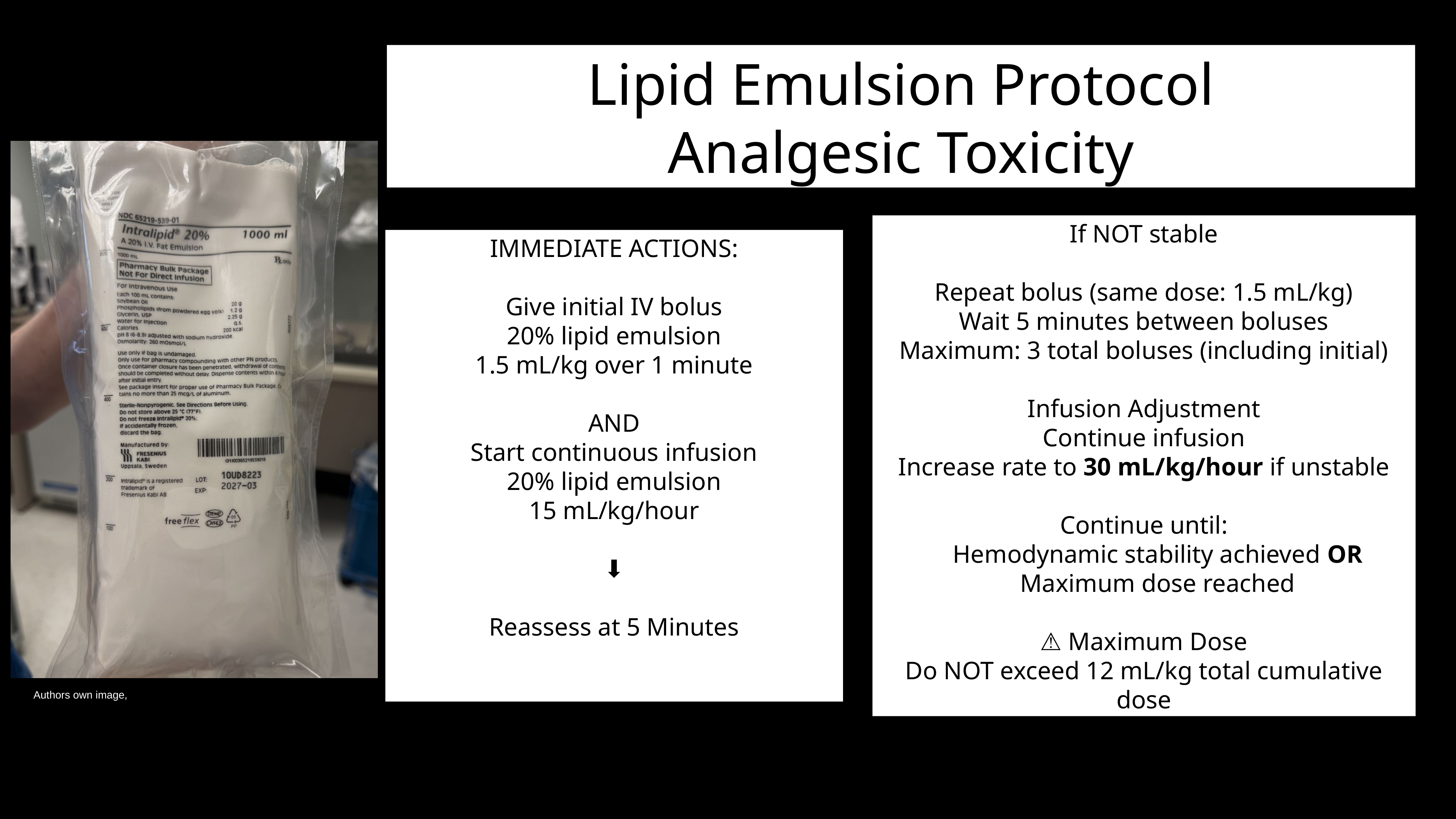

Lipid Emulsion Protocol
Analgesic Toxicity
If NOT stable
Repeat bolus (same dose: 1.5 mL/kg)
Wait 5 minutes between boluses
Maximum: 3 total boluses (including initial)
Infusion Adjustment
Continue infusion
Increase rate to 30 mL/kg/hour if unstable
Continue until:
Hemodynamic stability achieved OR
Maximum dose reached
⚠️ Maximum Dose
Do NOT exceed 12 mL/kg total cumulative dose
IMMEDIATE ACTIONS:
Give initial IV bolus
20% lipid emulsion
1.5 mL/kg over 1 minute
AND
Start continuous infusion
20% lipid emulsion
15 mL/kg/hour
⬇️
Reassess at 5 Minutes
Authors own image,

## Slide 21
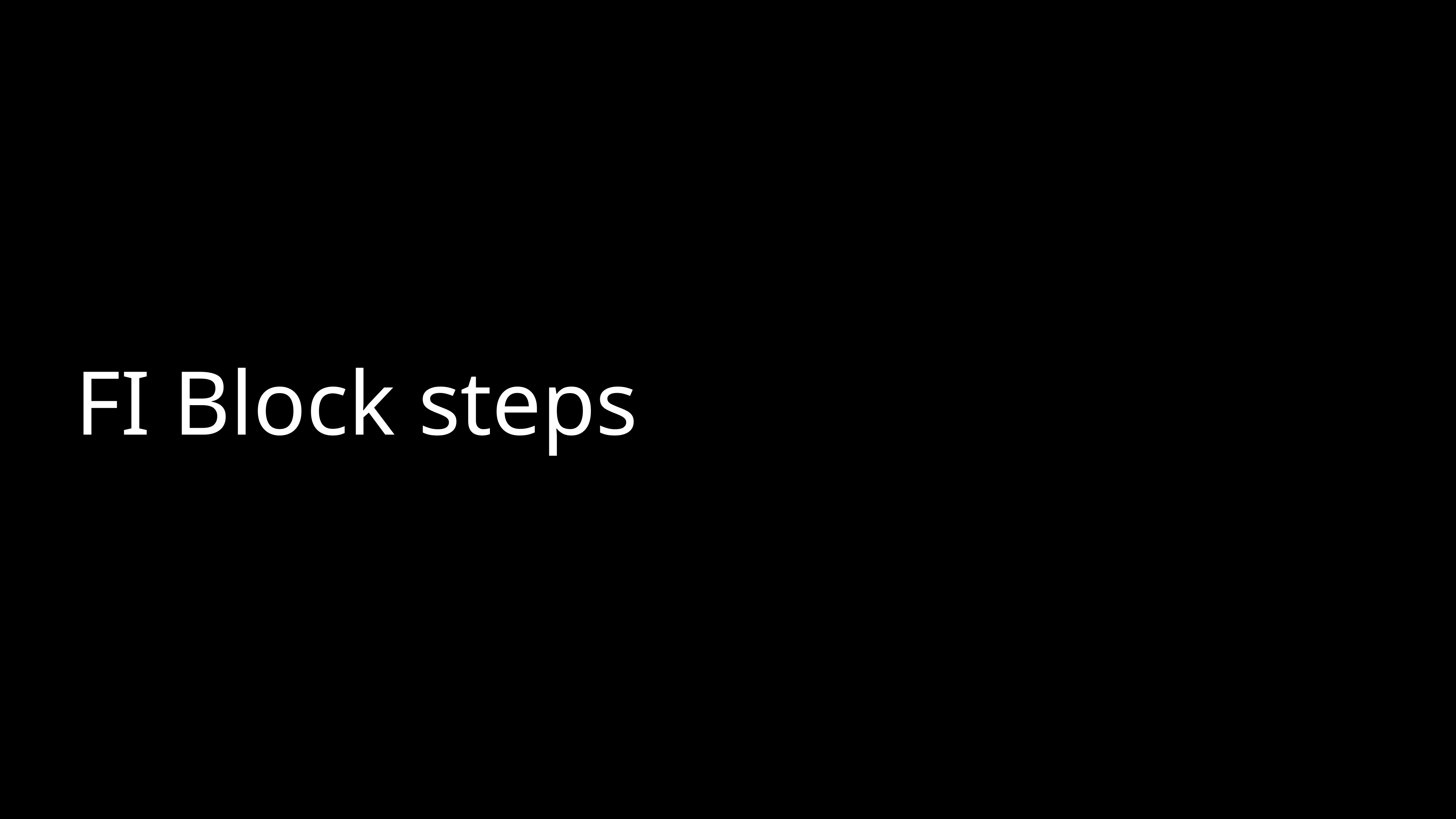

# FI Block steps

## Slide 22
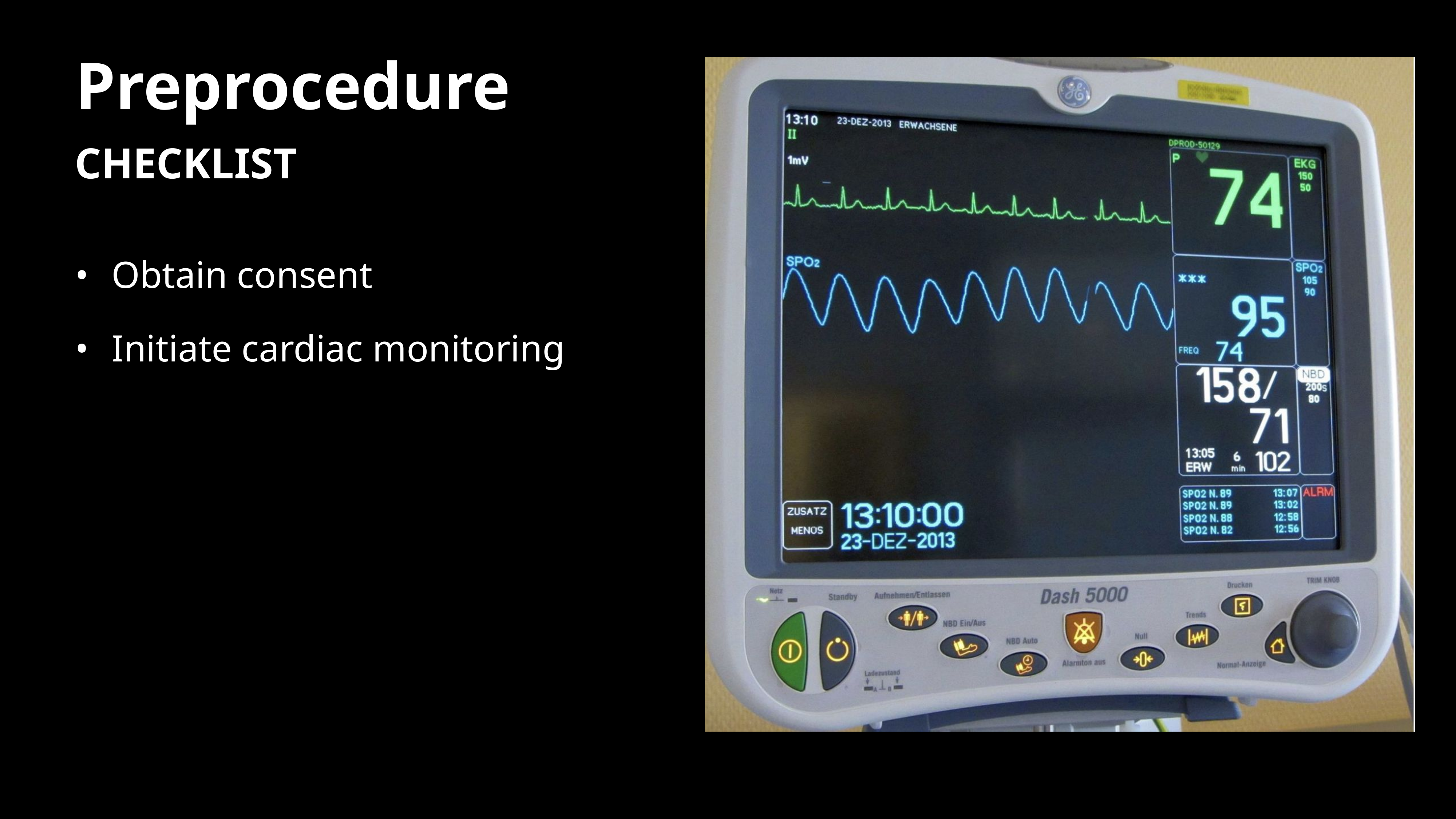

# Preprocedure
CHECKLIST
Obtain consent
Initiate cardiac monitoring
Image from Google stock images

## Slide 23
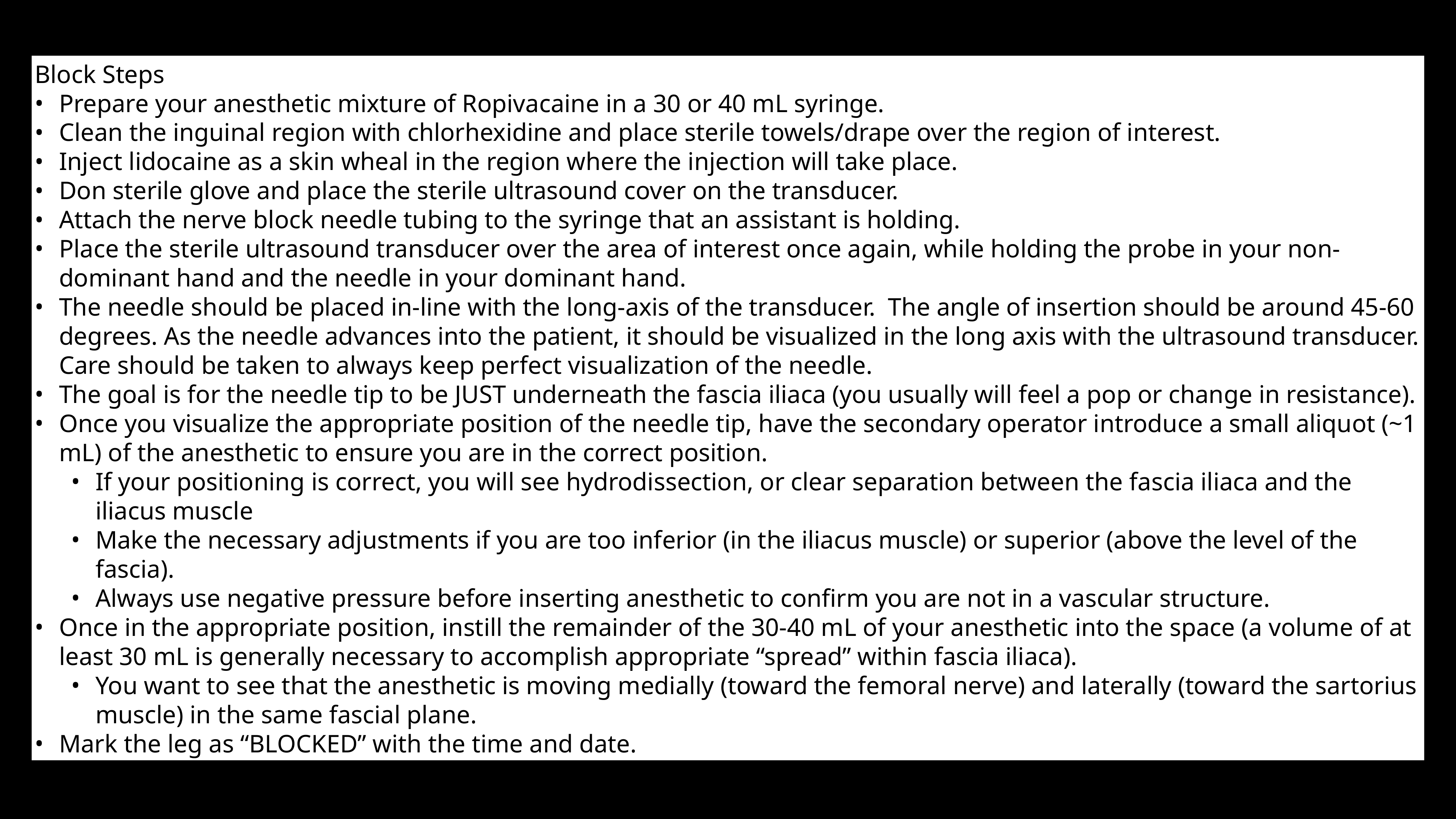

Block Steps
Prepare your anesthetic mixture of Ropivacaine in a 30 or 40 mL syringe.
Clean the inguinal region with chlorhexidine and place sterile towels/drape over the region of interest.
Inject lidocaine as a skin wheal in the region where the injection will take place.
Don sterile glove and place the sterile ultrasound cover on the transducer.
Attach the nerve block needle tubing to the syringe that an assistant is holding.
Place the sterile ultrasound transducer over the area of interest once again, while holding the probe in your non-dominant hand and the needle in your dominant hand.
The needle should be placed in-line with the long-axis of the transducer.  The angle of insertion should be around 45-60 degrees. As the needle advances into the patient, it should be visualized in the long axis with the ultrasound transducer. Care should be taken to always keep perfect visualization of the needle.
The goal is for the needle tip to be JUST underneath the fascia iliaca (you usually will feel a pop or change in resistance).
Once you visualize the appropriate position of the needle tip, have the secondary operator introduce a small aliquot (~1 mL) of the anesthetic to ensure you are in the correct position.
If your positioning is correct, you will see hydrodissection, or clear separation between the fascia iliaca and the iliacus muscle
Make the necessary adjustments if you are too inferior (in the iliacus muscle) or superior (above the level of the fascia).
Always use negative pressure before inserting anesthetic to confirm you are not in a vascular structure.
Once in the appropriate position, instill the remainder of the 30-40 mL of your anesthetic into the space (a volume of at least 30 mL is generally necessary to accomplish appropriate “spread” within fascia iliaca).
You want to see that the anesthetic is moving medially (toward the femoral nerve) and laterally (toward the sartorius muscle) in the same fascial plane.
Mark the leg as “BLOCKED” with the time and date.

## Slide 24
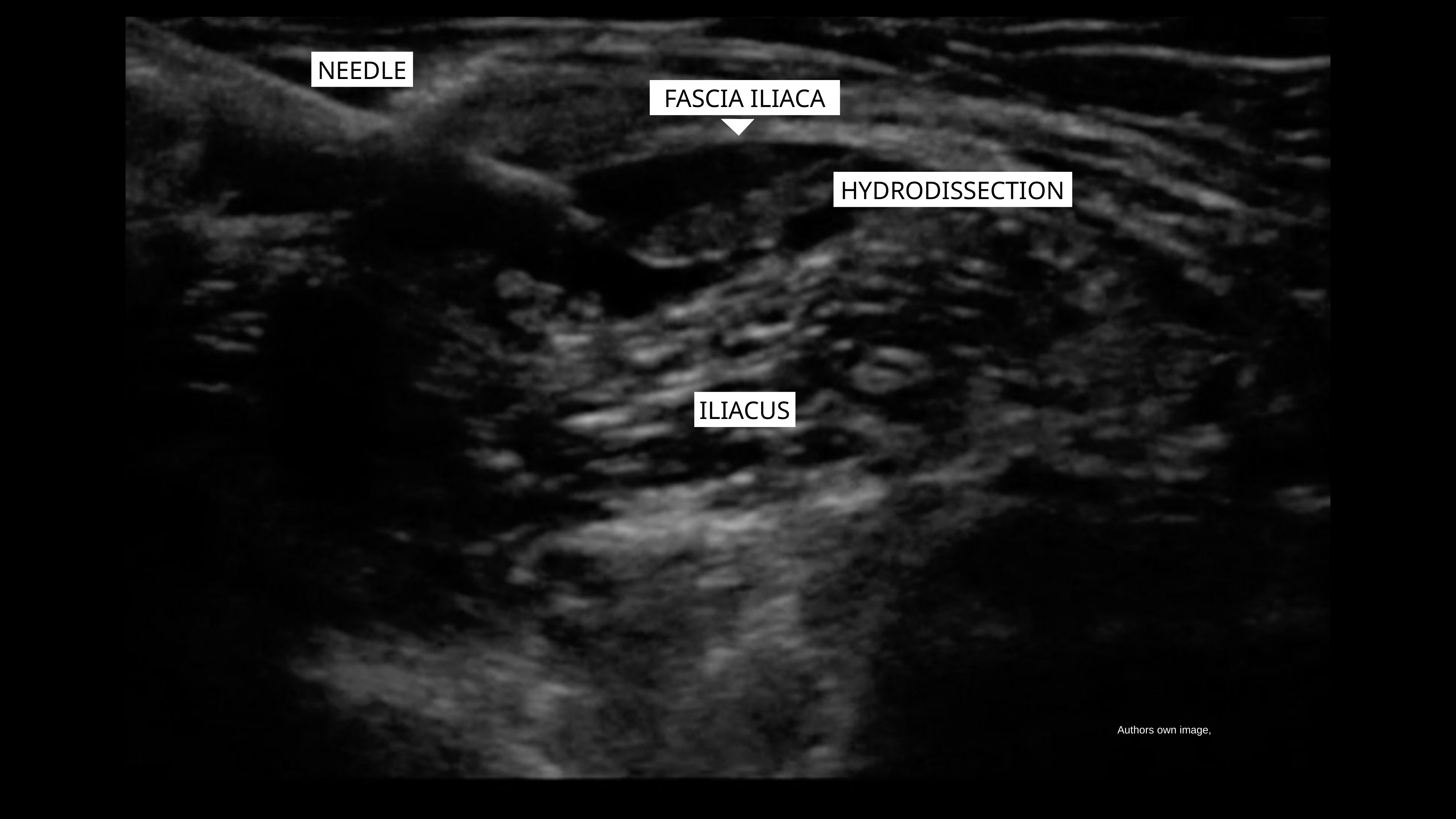

NEEDLE
FASCIA ILIACA
HYDRODISSECTION
ILIACUS
Authors own image,

## Slide 25
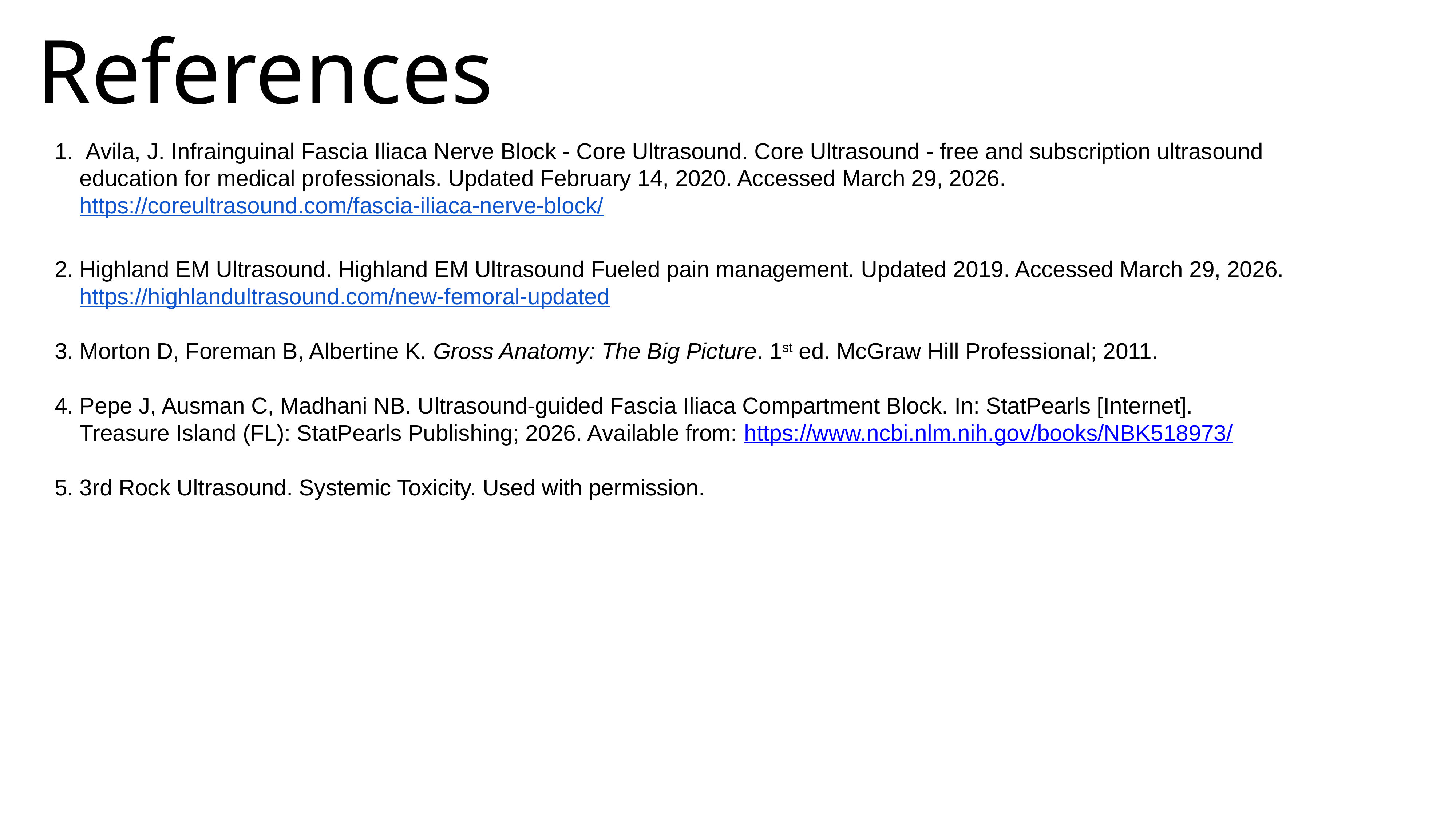

References
 Avila, J. Infrainguinal Fascia Iliaca Nerve Block - Core Ultrasound. Core Ultrasound - free and subscription ultrasound education for medical professionals. Updated February 14, 2020. Accessed March 29, 2026. https://coreultrasound.com/fascia-iliaca-nerve-block/
‌Highland EM Ultrasound. Highland EM Ultrasound Fueled pain management. Updated 2019. Accessed March 29, 2026. https://highlandultrasound.com/new-femoral-updated
‌Morton D, Foreman B, Albertine K. Gross Anatomy: The Big Picture. 1st ed. McGraw Hill Professional; 2011.
‌Pepe J, Ausman C, Madhani NB. Ultrasound-guided Fascia Iliaca Compartment Block. In: StatPearls [Internet]. Treasure Island (FL): StatPearls Publishing; 2026. Available from: https://www.ncbi.nlm.nih.gov/books/NBK518973/
3rd Rock Ultrasound. Systemic Toxicity. Used with permission.
